# A Global Survey on the Perception of Conservationists Regarding Animal Consciousness

**DOI:** 10.3390/ani15030341

**Published:** 2025-01-24

**Authors:** Valentine Yakhlef, Manuel Magalhães-Sant’Ana, Ana Luísa Pereira, Alexandre Azevedo

**Affiliations:** 1 CIVG—Vasco da Gama Research Center/EUVG—Vasco da Gama University School, Avenida José R. Sousa Fernandes 197, 3020-210 Coimbra, Portugal; ana.pereira@euvg.pt (A.L.P.); ax.c.azevedo@gmail.com (A.A.); 2 CIISA—Centro de Investigação Interdisciplinar em Sanidade Animal, Faculdade de Medicina Veterinária, Universidade de Lisboa, 1300-477 Lisbon, Portugal; mdsantana@fmv.ulisboa.pt; 3 Laboratório Associado para Ciência Animal e Veterinária (AL4AnimalS), 1300-477 Lisbon, Portugal; 4 CISAS—Center for Research and Development in Agrifood Systems and Sustainability, Escola Superior Agrária, Instituto Politécnico de Viana do Castelo, 4900-347 Viana do Castelo, Portugal

**Keywords:** animal mind, consciousness, conservation, human–animal relation, wildlife

## Abstract

Human perceptions of animal consciousness have evolved over time, influencing how they are treated. This study aimed to assess perceptions of animal consciousness in people working in conservation. An online survey of 87 participants evaluated their perceptions of animals’ capabilities across 10 dimensions of consciousness, including self-consciousness, learning, and reasoning. The sum of the answers to the questions was validated as a “perception index” using a principal component analysis. Next, its variation according to taxonomic, demographic, professional factors, and the duration, type, and frequency of interaction with animals was assessed with generalized linear models and stepwise model selection. Participants’ perceptions varied with taxonomic class and the level of education, with mammals obtaining higher indexes than birds (−0.14; 95% confidence interval [CI] −0.24, −0.03, *p* < 0.01) or reptiles (−0.41; 95% CI −0.55, −0.27, *p* < 0.01), and respondents holding a PhD exhibiting higher perception indexes than those with BScs (−0.19; 95% CI −0.32, −0.06, *p* < 0.01) or lower education (−0.18; 95% CI −0.32, −0.03, *p* = 0.01). The attribution of consciousness followed a phylogenetic pattern, but several exceptions (e.g., the octopus and raven) were noted on a finer scale supporting a multifactorial influence on the perception of animal consciousness that emerges upon a baseline phylogenetic pattern. Finally, the results suggest that conservationists are influenced by culture and scientific knowledge, as much as their personal experiences, when evaluating animals’ perceptions, highlighting the need for further research on the convergence between perception, belief, and evidence.

## Simple Summary

People’s attitudes toward wild animals spark discussions and influence decision-making in wildlife research and conservation. One of the strongest and most consistent predictors of attitudes toward animal use is whether people believe that animals have thoughts and feelings. This study looked at how conservationists view the mental abilities of the animals they work with. Responses from 87 participants followed a general pattern, where larger and more complex animals, like mammals, were seen as having more advanced mental abilities than smaller animals, like insects. However, some exceptions to this pattern were found, suggesting that other factors, such as an animal’s attributes, its role in human life, cultural influences, or scientific information, shape these perceptions.

## 1. Introduction

Humans’ perception of animals’ ability for consciousness has evolved over time. Aristotle was a vitalist, which is a philosophical stance asserting that living organisms have a life force distinct from physical processes; he thought that all living beings—plants, animals, humans—had souls and that “it is the possession of sensation that leads us for the first time to speak of living things as animals” (II.2, p. 413b) [1]. René Descartes, like Aristotle, believed that mind and body are separate entities, but contrary to the former, did not think that animals had souls or attributed the ability to experience feelings to animals (p. 516) [2]. In the eighteenth century, Immanuel Kant attributed primacy to humans but acknowledged their indirect duties towards other sentient beings, whereas the increasing recognition of animal consciousness became more prominent with the opposition to vivisection (for a detailed review, see Franco, 2013 [3]). The concept of animal sentience—the capacity for animals to experience feelings—was highlighted by illuminist thinkers such as Voltaire and Rousseau, but it was Bentham who forged the foundation of a utilitarian approach based on animal sentience that has remained influential to date: “The question is not, Can they reason? nor, Can they talk? but, Can they suffer?” [4]. Therein lies the view that animals need not communicate or possess cognitive abilities like humans to be considered moral subjects. If they are conscious and can experience feelings, their welfare needs to be considered. Since the end of the twentieth century, researchers have recognized the implications of sentience for animal welfare decisions [5], and the social recognition of animal sentience and consciousness fueled by growing scientific evidence is reflected in both the Cambridge and New York Declarations [6,7]. In these declarations, groups of scientists present simple and synthesized updates on animal consciousness to society. For example, the Cambridge Declaration focuses on the evidence indicating that the human neocortex is not the only structure associated with consciousness and that other animals, namely, “all mammals, birds, and octopuses”, possess the necessary structures for consciousness. This approach posits that animals possessing neuroanatomical structures analogous to those enabling human sentience, consciousness, and cognition likely share similar capabilities.

Several definitions are relevant to this discussion and have been used differently in the literature. In this study, cognition is distinguished from consciousness. Cognition encompasses mental processes such as perception, memory, and learning, which have evolved to help animals cope and interact with their external environment. Consciousness, on the other hand, represents an animal’s ability to be aware of subjective, affective, and perceptual experiences [8,9], enabling it to perceive its environment and experience subjective feelings such as pain or sadness. Finally, sentience is defined as the capacity to feel and perceive. The acknowledgment that animals possess different levels of consciousness and cognition raises even more questions about their treatment, and has influenced how humans treat animals for centuries [10]. To date, consciousness remains one of the most elusive and challenging aspects to study among all biological phenomena [11]. The ultimate objective for researchers exploring animal consciousness involves identifying which animal species possess consciousness, to what degree, and the nuances among them [8,12]. Recent studies by Birch and colleagues [8] addressed these different definitions by deconstructing them into specific capabilities that animals may or may not possess, referred to as dimensions of consciousness. This approach helps overcome the inherent anthropocentric bias of the definitions, by focusing on specific and objective capabilities of evolutionary and adaptive value. It also acknowledges the possibility that different species may exhibit distinctive types of sentience, consciousness, or cognition. Subsequently, other authors have further applied this tool using practical examples [12]. It is noteworthy that some of the dimensions considered may fall under the definitions of sentience or cognition. However, since this study focuses on individual dimensions and whether humans perceive animals as possessing them, the discussion of the hierarchical relations between the definitions falls beyond its scope. This approach provides objective definitions of dimensions of consciousness, making them easier to interpret and hence amenable to survey studies involving individuals without a scientific background.

Over the centuries, human attitudes toward animals have been shaped by an increasing belief in animal minds and evidence of animal consciousness. The inclusion of an increasing number of organisms in the sphere of bioethical consideration was coined by Singer as the “expanding circle” [13]. However, this belief is shaped by the animals’ species and tends to follow natural (scala naturae) [4] or sociozoological [14] scales, based on phylogeny or the roles animals play in human lives. Human attitudes toward wild animals influence decisions regarding resource allocation for their protection and conservation. Decisions are often biased by species “charisma”, favoring large mammals and birds, even when other species are more numerous, critical to ecosystem functioning, or threatened [15,16].

Changes in the attitudes of humans toward wild animals have spawned debate and influenced decision-making in wildlife research and conservation, with approaches that involve invasive interventions or lethal sampling methods becoming increasingly unacceptable to society [17,18,19]. Studies on how conservation decisions are influenced by the perception of how animals experience life are lacking. However, the few studies on domestic species indicate that the way people perceive animals’ capabilities for cognition and consciousness directly influences the way they are treated [20,21,22,23]. Additionally, research indicates that the belief in the animal mind, which is inherently related to perceptions of animal consciousness, is one of the strongest and most consistent predictors of attitudes toward animal use [24,25,26,27]. Most people have little contact with wild species, and their perception is subject to cultural differences, often influenced by myths, folklore [28], or primordial fears [29]. People working in the field of conservation are uniquely positioned to empirically assess the capabilities for consciousness of wild species. Global conservation efforts require that conservationists work closely with animals, observing their behavior and reactions firsthand. This cross-sectional study aims to explore how people working in both in-situ and ex-situ wildlife conservation perceive the consciousness of the animals under their care. Our first objective was to characterize the perception of caregivers on the capabilities for consciousness of the species under their care based on the dimensions proposed by Birch et al. [8] and Dung and Newen [12]. Our second objective was to assess how those perceptions varied with species, demographic factors, and the duration and type of their interaction with the animals. Drawing on previous research on human attitudes toward animals, we predicted that the perception of animal consciousness would vary with respondent age and gender [30,31,32] and the level of familiarity with the animals [27], but we also expected it to vary with the geographical location, level of education, and area of professional training of respondents in animal-related professions.

## 2. Materials and Methods

A cross-sectional study was conducted using a survey hosted on the Google Forms platform. The survey was composed of four sections. The first consisted of demographic questions, while the second used a Likert scale ranging from 1 (“*completely disagree*”) to 5 (“*completely agree*”) to assess participants’ perception of the consciousness of a single species they most frequently worked with, based on the dimensions of animal consciousness proposed by Dung and Newen [12]. A detailed description of the development of the questions is available in the Supplementary Materials (File S1). Given the complexity of the questions, respondents were not required to answer them to proceed to the next section of the survey. The third part concerned the human–animal relation, with multiple-choice questions about the type and frequency of interaction respondents had with the animals. Finally, the last part consisted of a multiple-choice grid, aimed at assessing participants’ perception of animal consciousness in predefined species, selected to include all classes, pests, or useful species, predators, and prey, and those with a reputation of high cognitive capabilities. The species selected were the housefly (*Musca domestica*), cockroach (*Blattella germanica*), tree frog (*Agalychnis callidryas*), Atlantic salmon (*Salmo salar*), honey bee (*Apis mellifera*), ball python (*Python regius*), house sparrow (*Passer domesticus*), rat (*Rattus norvegicus*), leopard (*Panthera pardus*), common octopus (*Octopus vulgaris*), wolf (*Canis lupus*), raven (*Corvus corax*), bottlenose dolphin (*Tursiops truncatus*), African elephant (*Loxodonta africana*), and the chimpanzee (*Pan troglodytes*). The response scale ranged from 1 (“*has no consciousness*”) to 7 (“*has consciousness comparable to humans*”).

Before dissemination, the survey was piloted by two veterinarians not involved in the study and chosen for their relevant expertise: one specializing in animal welfare and the other experienced in wild and exotic animals, whose suggestions for improvement were considered. The Google Forms survey link was directly emailed between 20 December 2023 and 15 March 2024 to conservation centers and veterinarians working in conservation (*n* = 156) across the world, which were selected by non-probability sampling using internet searches followed by snowball sampling through shared contacts. The full questionnaire is provided in the Supplementary Materials (File S2).

### 2.1. Data Preparation

Data cleaning was performed using Google Sheets. For statistical modeling, participants’ professions were grouped into predefined categories: “*biologist, ecologist or conservationist*”, “*ethologist*”, “*keeper, caretaker or rehabilitator*”, “*veterinarian*”, “*veterinary technician or veterinary nurse*”, and “*other*” (which included, for example, engineers, PhD students, and attorneys). Regarding the education level, due to the low number of responses, the options “*primary education*” (*n* = 1), “*lower secondary education*” (*n* = 2), and “*upper secondary education*” (*n* = 14) were grouped into the category “*lower education*” (*n* = 17). For each species chosen by participants, taxonomic levels (family, order, and class) were established (see Table S5). The widely recognized Linnean taxonomic classification was used to ensure that respondents could easily identify and categorize animals when assessing their consciousness. For example, while birds are phylogenetically part of the Reptilia clade [33], they are presented separately from reptiles to reflect the classification most familiar to a general audience. The duration of the participants’ work with the target animal species was converted into years and rounded up. A numerical value was assigned to each response to the question regarding the frequency [ranging from 0 (“*never interact*”) to 4 (“*interact on a daily basis*”)] and the type [ranging from 0 (“*no interaction*”) to 6 (“*surrogate parenting for at least a period of the animal’s life*”)] of interactions between participants and the animal of their choice. The responses where participants chose multiple species or did not provide specific species names (*n* = 5) were included in the sample demographics but excluded from descriptive and inferential analyses. To obtain a single dependent variable that encompassed the overall perception of each participant on the consciousness capabilities, a “*perception index*” was created by calculating the sum of the answers to all of the Likert scale questions from that respondent. Since the relevance of each dimension of consciousness is unknown and thought to be heterogeneous among species [12], equal weighting of each consciousness dimension was assumed for the calculation of this index. Because of this limitation, cautious interpretation of the results of this index is required, which should be limited to assessing the effect of the influence of respondent factors on the perception results. Since the possible answers ranged from 1 to 5, the perception index resulted in a discrete variable of count data, ranging from 28 (answering 1 to all questions) to 140 (answering 5 to all questions).

### 2.2. Statistical Analysis

Descriptive statistical analysis was conducted using Google Sheets. Scores for each dimension of consciousness were calculated by averaging the scores of all responses for each question, and then calculating the average score for the dimension (see Supplementary Materials (Tables S6 and S7)). Radar diagrams adapted from the approach of Dung and Newen [12] were created to visualize respondents’ perceptions of the consciousness abilities of each taxonomic group. The dimensions were arranged alphabetically for consistency. A principal component analysis was employed to investigate the validity of the *perception index* as a single variable representing the participant’s perception of animal consciousness. The average of perception index was calculated for each taxonomic class, order, and family and illustrated in bar plots.

We performed analyses to determine which factors influence participants’ perception of consciousness in the species they work with using R version 4.0.3 [34]. For this purpose, we analyzed the influence of relevant factors on the perception index using generalized linear models (GLMs). First, we fitted a GLM with all candidate variables based on their biological relevance. Candidate variables included respondents’ residence (grouped by continent), age, gender, level of education, profession, number of years working with the species, taxonomic class of the species, frequency of their interaction with the animals, and type of interaction with the animals. We tested for multicollinearity using the variance inflation factor, with a cut-off value of 6, which led to the exclusion of two variables (location and the frequency of interaction with the animals). We checked the models for overdispersion with the function *dispersiontest* of the AER package [35]; the negative binomial distribution performed better that Poisson or quasi-Poisson distributions, and was hence selected for model fitting. Next, we performed stepwise model selection to obtain the most parsimonious model using the *stepAIC* function from the MASS package [36] and validated model assumptions through the visual inspection of QQ-plots and residuals vs. fitted plots, and comparisons of observed vs. simulated counts (posterior predictive checks). Finally, we presented the results of the final model as estimates, *p*-values, and 95% confidence intervals, and illustrated them in forest plots.

## 3. Results

A total of 92 responses were obtained (out of 156 invitations), corresponding to a return rate of 59.0%. After data cleaning, five responses were excluded from the analysis as the respondents referred to multiple species when answering the questionnaire, leaving a final sample of 87. A summary of the survey demographics can be found in the Supplementary Materials (Tables S2–S4). Responses were gathered from 30 nationalities across five continents (with a greater representation of Europe and America). Participants identified themselves as biologists, ecologists, or conservationists (35.6%), followed by veterinarians (27.6%) and keepers, caretakers, or rehabilitators (25.3%), ensuring a balanced representation of the professions usually involved in conservation. Responses were obtained from people with varied levels of education and age classes, with a predominance of females (63.2%).

**Figure 1:**
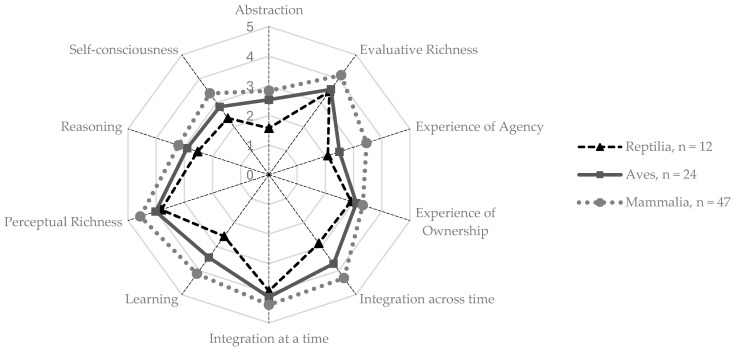
Average agreement scores regarding the capabilities for consciousness for each dimension calculated for each taxonomic class (classes with *n* > 1).

The respondents generally agreed that all species represented in the survey possessed capabilities of perceptual and evaluative richness, integration at and across time, and learning, with average scores between 3.71 and 4.30 (Supplementary Materials (Table S7)). The dimensions of experience of ownership, self-consciousness, reasoning, and experience of agency received average scores near 3 (*neither agree nor disagree*). Only the dimension of abstraction received average scores indicating disagreement (2.56). Among taxonomic classes, mammals scored highest in perceived consciousness, followed by birds, and then reptiles (Figure 1). Insects (e.g., honeybees or houseflies), bony fish (e.g., salmon or seabass), cartilaginous fish (e.g., sharks and rays), and cephalopods (e.g., octopus or squid) each received only one response, with notably high scores (5.0) for the dimensions of integration at a time for insects and perceptual richness for cartilaginous fish.

**Figure 2:**
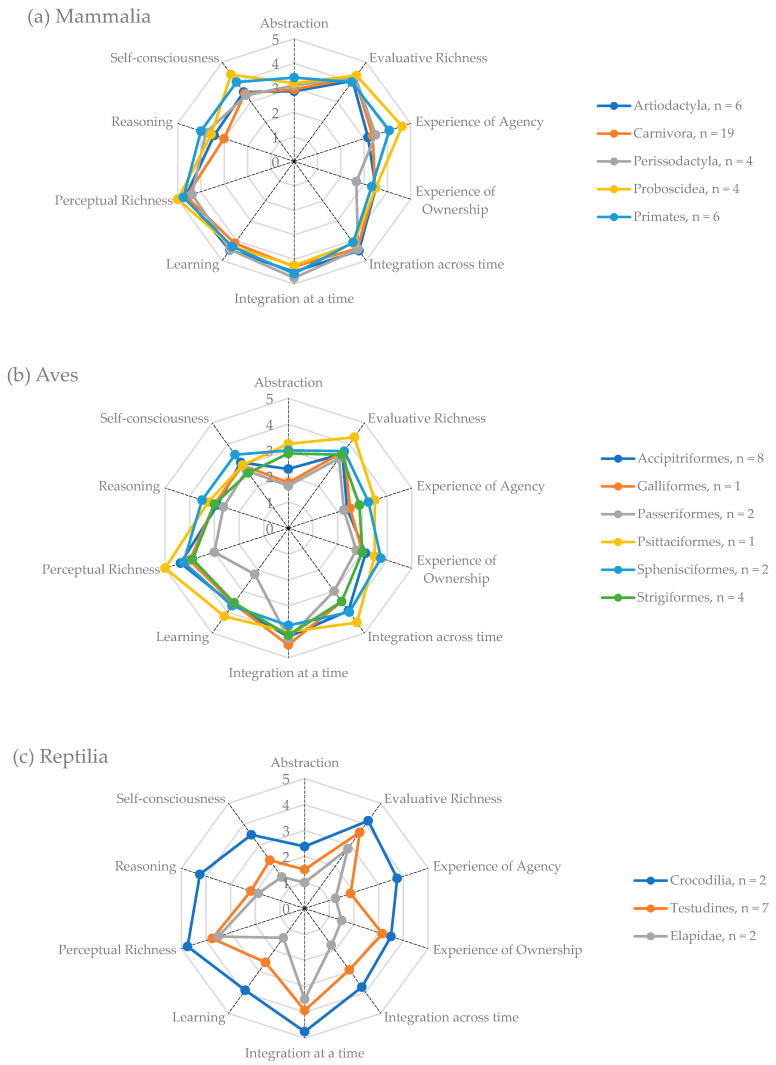
Average agreement scores regarding the capabilities for consciousness for each dimension calculated for each selected taxonomic groups within the classes of mammals (**a**), birds (**b**), and reptiles (**c**).

Among mammals, elephants (*Proboscidea*) scored the highest in the dimensions of perceptual and evaluative richness, experience of agency, and self-consciousness. However, primates scored highest in reasoning and abstraction, while odd-toed ungulates (e.g., zebras, rhinoceros, or tapirs) scored highest for integration at and across time (Figure 2a). Within birds, differences in the agreement scores were noted between different avian groups, with psittacine birds (e.g., parrots and macaws) obtaining higher scores in the dimensions of perceptual and evaluative richness and integration across time, and penguins (*Sphenisciformes*) achieving the highest agreement scores in the dimensions of self-consciousness and reasoning (Figure 2b). Finally, among reptiles, clear differences regarding agreement scores were found between groups, with higher scores being attributed to crocodilians, followed by chelonians (turtles and tortoises), and finally snakes (Figure 2c).

### 3.1. Consciousness Perception Index

The validation of the perception index was performed using principal component analysis. After the reduction of the dimensionality of the data and the transformation of the original variables (28 questions) into a set of uncorrelated variables (principal components), 41.1% of the total variance was explained by the retained components, indicating that a substantial portion of the variability was captured. The first principal component closely mirrors the overall perception index, with an eigenvalue of 15.82, accounting for 38.5% of the total variance. The second eigenvalue obtained was 3.68, representing 8.9% of the total variance. These results indicate that the index captures a substantial portion of the variability in the data.

**Figure 3:**
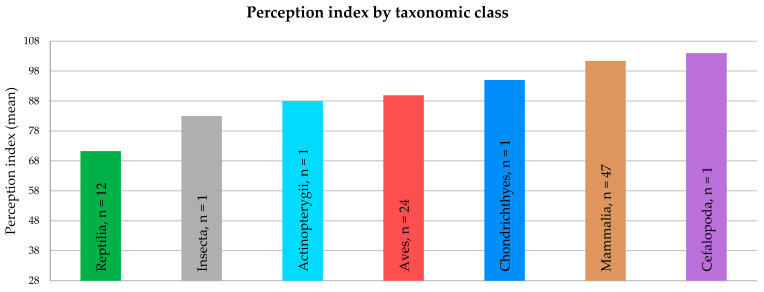
Distribution of the average animal consciousness perception index (range 28 to 140) according to the taxonomic class of the species considered by each respondent.

The average perception index differed between taxonomic classes (Figure 3). Among the classes with multiple observations in the sample, mammals scored highest on the index, followed by birds, and finally reptiles. The score of cephalopods (in this specific case, the common octopus) was the highest of all; cartilaginous fish had the third highest score, and insects scored higher than the average score for reptiles. However, these classes each had only a single observation, so these results are descriptive and should be interpreted with caution. The average scores of perception index by order and family can be found in the Supplementary Materials (Figures S3 and S4).

### 3.2. Factors That Influence the Perception Index

**Figure 4:**
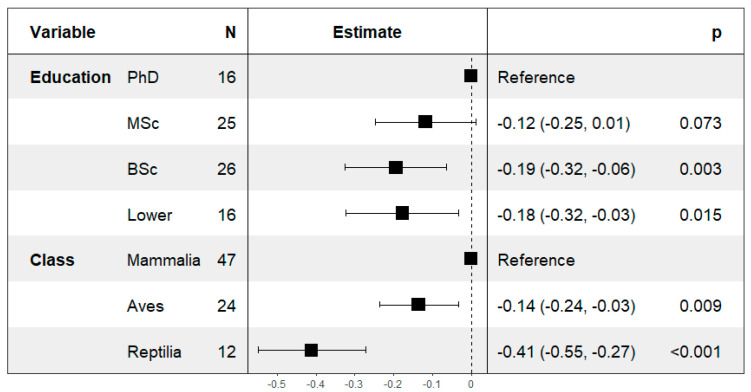
Results of the final (most parsimonious) generalized linear model explaining the variance in the animal consciousness perception index. Results are presented as estimates, 95% confidence intervals and *p*-values. For each factor, the variable level associated with higher perception indexes was chosen as the reference level.

The assessment of the influence of different factors on the overall perception of consciousness that each respondent attributed to the species was performed using GLM and stepwise model selection. The variables retained in the most parsimonious model to explain the variation in the perception index included the animal class and the level of education (Figure 4). The other candidate variables (continent of residence, age, gender, profession, number of years working with the species, frequency, and type of interaction with the animals) did not improve model parsimony and were therefore considered not to influence the variation in the perception index data. Respondents with a PhD did not differ significantly from those with a MSc, but attributed significantly higher perception indexes when compared to those with a BSc (−0.19; 95% CI −0.32, −0.06, *p* < 0.01) and lower education (high school or less) (−0.18; 95% CI −0.32, −0.03, *p* = 0.01). In terms of animal class, people working with mammals attributed a significantly higher perception index to the animals they work with when compared to those working with birds (−0.14; 95% CI −0.24, −0.03, *p* < 0.01) and reptiles (−0.41; 95% CI −0.55, −0.27, *p* < 0.01). Finally, respondents working with birds also attributed higher perception indexes when compared to those working with reptiles.

### 3.3. Perceptions of Respondents on Predefined Species

**Figure 5:**
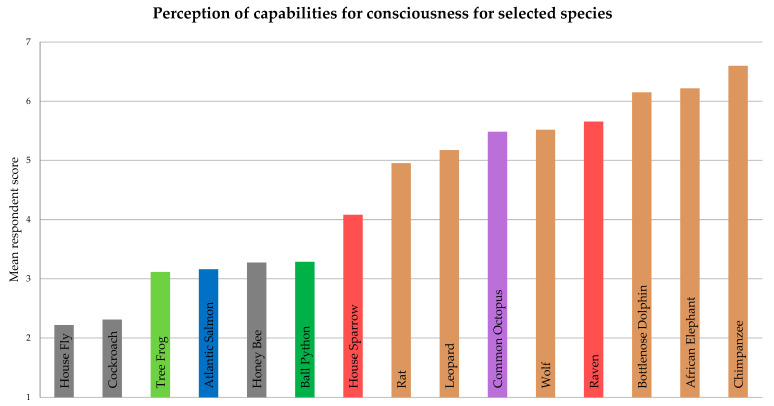
Average scores of respondents’ perception of capabilities for consciousness for selected species (presented as means), on a scale from 1 (“*has no consciousness*”) to 7 (“*has consciousness comparable to humans*”). Colors of the bars represent the taxonomic class: brown for mammals, purple for cephalopods, red for birds, gray for insects, blue for fish, dark green for reptiles, and light green for amphibians.

The average scores obtained regarding the perception of capabilities for consciousness of predefined species showed clear differences between them (Figure 5). On a scale of 1 to 7, where a consciousness comparable to humans would correspond to a score of 7, the chimpanzee (6.6), elephant (6.2), and dolphin (6.2) obtained the highest scores. The highest score among non-mammalian animals was obtained by the raven (5.7), which was followed by the wolf (5.5) and then another non-mammalian species (a cephalopod), the common octopus (5.5). The leopard (5.2), rat (4.9), and house sparrow (4.1) followed with scores above the center point of the scale, after which the ball python (3.3), honeybee (3.3), salmon (3.2), tree frog (3.1), cockroach (2.3), and housefly (2.2) had scores below 4.

## 4. Discussion

This study aimed to gauge the perception of professionals involved in conservation regarding the dimensions of consciousness of the animal species with which they work. As humans, our perception of animals’ capabilities for consciousness influences how we treat them. The results of our survey indicate that the perception of animals’ capability for consciousness is influenced, at a broad level, by the animals’ taxonomic class and the person’s level of education. However, at the species level, subtle variations are likely due to factors beyond the taxonomic and phylogenetic characteristics or the respondents’ demographic variables. Regarding the factors that influence the perception of animals’ capabilities for consciousness among conservationists, it is noteworthy that respondent age and gender were not retained in the final models. This is unexpected because these factors often influence perceptions and attitudes towards animals [30,31], including wildlife [32]. The number of years working with the species, the profession, and the frequency and type of interaction with animals are associated with differences in familiarity with the species, ability to interpret its behavior, and different bonds with individual animals. Therefore, they were expected to influence human perceptions of animal consciousness [27]. However, these factors were also excluded from the most parsimonious model and further research is required to help explain these results. While different professions were surveyed (e.g., veterinarians, biologists, and keepers), a common professional culture among those involved in conservation could bias the statistical effect associated with the area of professional training. For example, a discourse analysis study concluded that some hunting organizations in Finland and the UK describe animals in a way that underestimates animal minds [37], which illustrates how industry culture might affect the perception of animal consciousness. Similarly, attitudes toward animals are likely to be influenced by the beliefs associated with working in conservation, as was observed in a study on the values and attitudes toward prairie dogs, where conservationists displayed the highest regard toward the animals among the sampled groups (ranchers, rural residents, and urban residents) [38].

In general, the attribution of dimensions of consciousness followed what could be the expected pattern based on a phylogenetic scale (scala naturae), increasing gradually from insects to mammals [24,39]. Closer examination at a finer taxonomic scale reveals notable exceptions. For example, psittacine birds (Psittaciformes) and penguins (Sphenisciformes) obtained higher perception ratings than other birds (Figure 2b) when assessed by the people working with them. Possible explanations for these differences include the influence of available scientific evidence, cultural factors, sociozoological factors, animal attributes, or characteristics of the respondents not captured in this study. The perception that a species is able to communicate has been associated with more empathic attitudes toward animals [40], which might explain the scores for psittacines, as well as a widespread reputation for being “intelligent” [41]. In the case of reptiles (Figure 2c), crocodilians obtained higher scores than chelonians and elapids (squamate reptiles), which, under the assumption that crocodilians and reptiles have earlier phylogenetic origins, seems to contradict the general trend. However, more recent studies place crocodilians closer to birds in phylogenetic terms [42]. Additionally, crocodilians can be perceived as more closely related to humans compared to squamates, particularly snakes, due to the presence of limbs and other anatomical features such as a four-chambered heart. Phylogenetic relatedness to humans has been associated with empathic attitudes toward animals [40]. In the case of snakes, limblessness and distinct patterns of behavior and communication could lead to their perception as being more distant from humans than they phylogenetically are.

One survey question required respondents to rate the consciousness of several predefined species (Figure 5). This offers further insight into variations, as the answers are not limited to the species the participants work with, and the selected species have particularities that differ from others of the same class. In this question, a departure from the scala naturae is observed in some cases, notably the raven and the octopus. The case of the raven is particularly interesting considering that it is included in the order Passeriformes, just like the house sparrow. However, both science and media have contributed to a reputation of it being an intelligent species [43,44], which can shape general perceptions, including those of conservationists. A similar case can be made for the octopus, a member of the coleoid cephalopods, considered the most cognitively advanced group of invertebrates [45]. As with the raven, its position in the results likely reflects the combined effect of scientific research and popular media. The order of these species within the results suggests that respondents might rely on such widely disseminated information, as much as their personal experience, when evaluating animals’ perceptions. Another exception to the scala naturae is apparent in the placement of the honeybee above the tree frog and the salmon, and comparatively higher than the other insects (Figure 5). While the octopus has been a target in cephalopod sentience, consciousness, and cognition research, the bee has been one of the target species for insects [46]. However, an additional explanation that could influence these results is the “cultural inheritance” [47] of the sociozoological scale, with human attitudes towards animals being influenced by the attribution of the status of “good” or “bad” according to their relation with humans as pets, vermin, predators, or tools [14]. This could justify the different scores attributed to bees compared with cockroaches or house flies. Another justification could be found in the physical attractiveness of bees compared to the other insects. Physical attractiveness, larger size, and resemblance to humans were shown to increase support for a species’ protection [48]. However, the comparatively high scores of the wolf suggest an alternative explanation, based on the beliefs of the specific group of respondents. Both bees and wolves are species with populations under threat and afforded conservation protection, while cockroaches and house flies are not, which might shape the way they are perceived and valued by conservationists.

When considering how we use and treat animals, it is worth discussing how our perception of their capabilities for consciousness aligns with their actual capabilities. An exploratory approach to this discussion can be attempted by comparing the results of our study with those of Birch et al. [8] and Dung and Newen [12]. Birch et al. established dimension-based consciousness profiles for elephants, corvids, and cephalopods. The results obtained for elephants in our study align closely with the evidence-based consciousness profiles from Birch et al., with one notable exception: the dimension of perceptual richness. The respondents in our study seem to overestimate this dimension compared to the limited visual and tactile perceptual richness of elephants [8]. Similarly, for cephalopods, the results are generally consistent between the two studies. However, our respondents overestimate the capabilities of integration at a time and self-consciousness compared to the limited evidence for these capacities in cephalopods [8]. The overall alignment between the perceptions of conservationists and the evidence-based profiles of the species suggests that conservationists, likely informed by both scientific knowledge and personal experience, have a nuanced understanding of the animals under their care. Instances of overestimation may result from an anthropomorphic perspective—an expected bias given that human consciousness is the only example we directly experience. In the absence of concrete evidence, respondents may attribute capabilities to animals, such as elephants, which reflect their own human perceptions, such as attributing high perceptual richness. Perceptions and attitudes toward animals are likely shaped by multiple factors. These include the animals’ attributes, the characteristics of the humans interacting with them, their utility to humans, cultural influences, and available scientific knowledge [49]. The results of this study among conservation professionals seem to support the effect of this multifactorial influence upon a baseline perception guided by a phylogenetic scale.

The greatest challenge of this study was to elaborate understandable questions to represent the operationalization of each dimension of animal consciousness. Despite the authors’ best efforts, the complexity of the questions could hinder respondents from answering the survey, with the risk of biasing the results, for example, regarding the effect of the level of education. Since this research targets a specific group of people working in an area based on the belief that animals are worth conserving, the results should not be generalized to other populations.

## 5. Conclusions

Perceptions of animals’ capabilities of consciousness among conservationists varied with the animals’ taxonomy—increasing from reptiles to birds and mammals—and the respondents’ level of education. However, variations were observed, with some species obtaining higher scores than others within the same taxon, such as psittacines or penguins among birds, and crocodilians among reptiles. While certain dimensions of consciousness (e.g., perceptual richness in elephants and integration at a time and self-consciousness in cephalopods) were occasionally overestimated, the general alignment of conservationists’ perceptions with scientific evidence is notable. These findings suggest that conservationists possess an informed perspective that likely reflects exposure to relevant knowledge, culture, and experience. Recognizing the established links between perceptions of animal consciousness and conservation priorities, our findings underscore the need for further research to guide effective decision-making in conservation. Future studies should explore how conservationists’ perceptions compare to those of other groups (e.g., the general public), their alignment with scientific evidence, and their influence on resource allocation and conservation practices.

## Data Availability

The original contributions presented in the study are included in the article/Supplementary Materials, further inquiries can be directed to the corresponding author.

## Supplementary Materials

The following supporting information can be downloaded at https://www.mdpi.com/article/10.3390/ani15030341/s1: File S1—Background information used to derive questions on the capabilities in each dimension of consciousness; Table S1—Summary table of the dimensions of consciousness, the investigation methods used, the questions, and examples included in the survey; File S2—Full questionnaire; Tables S2–S4—Demographic characterization of the survey sample; Table S5—Taxonomic class, order and family of the animals participants choose to respond about; Tables S6 and S7 and Figures S1 and S2—Average Likert scale scores for each question and dimension of animal consciousness; Table S8—Distribution of the averages and STDEV obtained for each dimension of animal consciousness from which questions are derived according to taxonomic classification; Table S9, Figures S3 and S4—Perception index according to taxonomic class, order, and family. References [8,12,50,51,52,53,54,55,56,57,58,59,60,61,62,63,64,65,66,67,68,69,70,71,72,73,74,75,76,77,78,79,80,81,82,83,84,85,86,87,88,89,90,91,92,93,94,95,96,97,98,99,100,101,102,103,104,105,106] are cited in the Supplementary Materials (File S1).
