# Supplementary material for: A Global Survey on the Perception of Conservationists Regarding Animal Consciousness
Source: Animals (Basel). 2025 Jan 24;15(3):341. doi: 10.3390/ani15030341 (PMC11816229; doi:10.3390/ani15030341)
Supplement: Supplementary file 1 [file animals-15-00341-s001.zip › Figure S4.pdf]

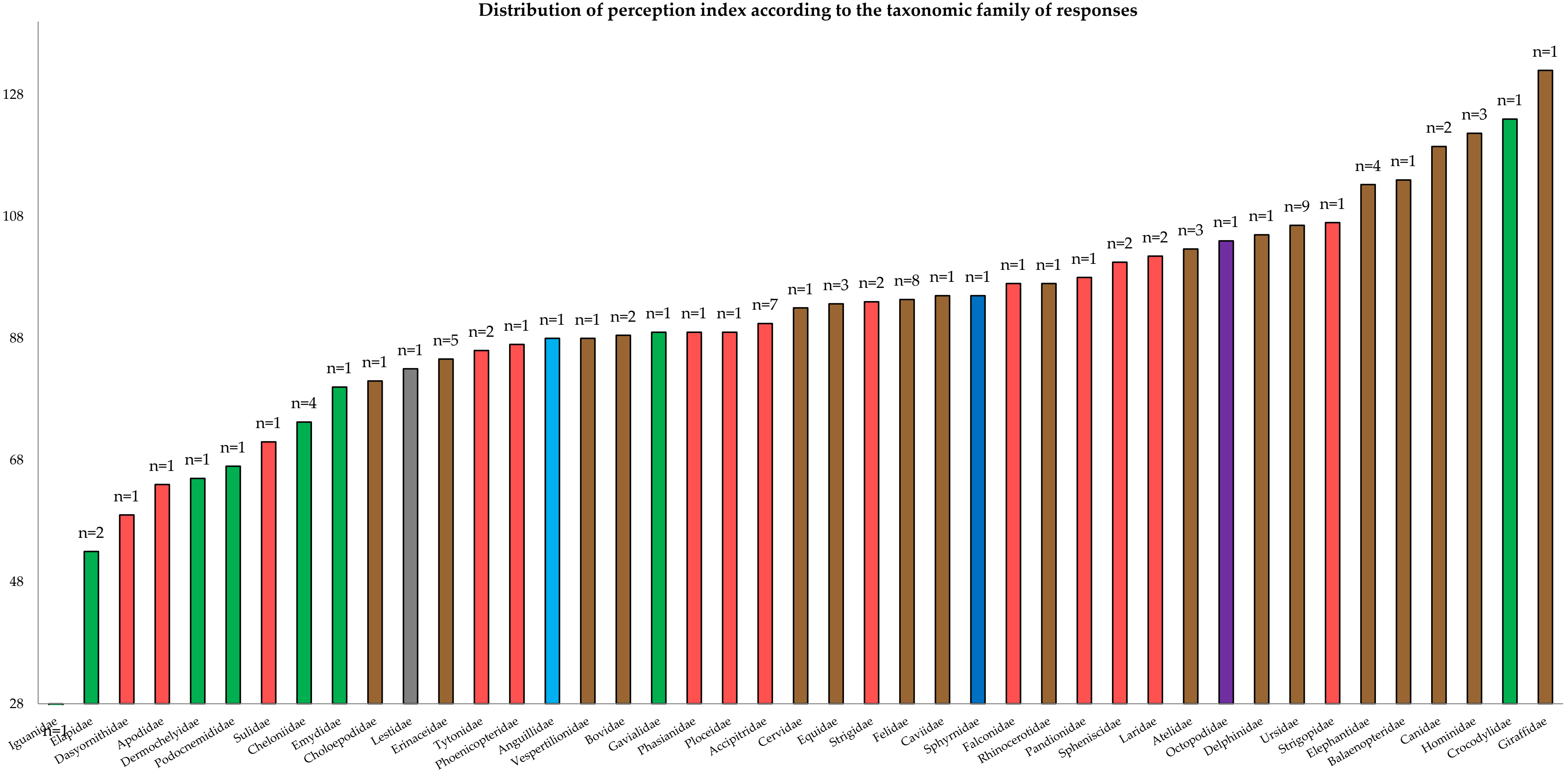

Figure S4. Distribution of perception index according to the taxonomic family of responses. Colors of the bars represent the taxonomic class: brown for mammals, purple for cephalopods, red for birds, grey for insects, blue for fish, dark green for reptiles.
