# Supplementary material for: A Global Survey on the Perception of Conservationists Regarding Animal Consciousness
Source: Animals (Basel). 2025 Jan 24;15(3):341. doi: 10.3390/ani15030341 (PMC11816229; doi:10.3390/ani15030341)
Supplement: Supplementary file 1 [file animals-15-00341-s001.zip › File S1.pdf]

File S1

Birch and colleagues (2020) and Dung and Newen (2023) have both proposed frameworks for evaluating animal consciousness across multiple dimensions. Their approaches serve as the starting point for our study on dimensions of animal consciousness. To build upon their work, we will further explore the literature these authors utilized to further define and develop these dimensions.

1- Perceptual-Richness

Perceptual-Richness refers to how animals perceive their environment, specifically focusing on the level of detail in their perception [1]. This dimension of consciousness can be explored through "perceptual categorization", "non-conceptual multisensory integration", "cross-modal learning" and "discrimination learning" [1].

Perceptual categorization refers to the ability of living beings to classify the elements in its surroundings into distinct categories [2] by recognizing the physical appearance of these elements [3]. In the first-place animals learn to recognize a specific element and generalize this knowledge to other elements [2]. In the light of this information, we can formulate the following representative survey question: *"Do you think that the animals under your care can recognize elements in their surroundings and categorise them?"** (Example: They see a keeper and to are able to classify him into the category "Human", or to see an insect, a fruit, a bottle of milk or a meat, and to classify it into the category "Food")*

No article was found including the definition of the term "non-conceptual multisensory integration"

Cross-modal learning consists of combining information from different senses to improve learning in each of them [4]. This allows animals to strengthen their memory, extract the logical structure of the world, facilitate perception and react quickly to events [5]. Based on this knowledge, a representative survey question could be: *"Do you believe that the animals under your care are capable of enhancing their learning across multiple senses by associating information perceived through different senses?"* (Example: We present images of food to an animal along with a corresponding smell. The animal associates the image with the scent, so when it is searching for food and detects the learned odor, it knows that the associated food can be found).

Discrimination learning represents the ability for an animal to learn to react differently depending on whether it's a rewarded stimulus or an unrewarded stimulus [6]. The type of discrimination learning can be auditory [7], visual [8-9-10], olfactory [6-11] or texture mediated [10]. With this information a representative survey question could be: *"Do you believe that the animals under your care are able to react differently depending on whether it's a rewarded stimulus or an unrewarded one?"**. (Example: Animals might react differently to a husbandry cue depending on whether or not you are holding a food reward item)*

2 - Evaluative-Richness

It is the ability for an animal to evaluate changes in internal or external states. This dimension is related to the emotional states of animals [12]. Evaluative-Richness can be explored through behaviours like

motivational trade-offs, play, judgment biases, and preference testing [1]. From this dimension we can formulate the following representative question: *"Do you believe that the animals under your care perceive and evaluate modification in their own internal or external states, and respond accordingly?"** (Example: They perceive the change in temperature when they arrive in a cold or hot place and they perceive the change in their emotions when they move from well-being to sadness)*

Motivational trade-off (behaviour) tests the capacity for an animal to compromise between two choices [13]. In this test, an uncomfortable stimulus is applied to an animal [13]. We see if the animal prefers to endure the uncomfortable stimulus in order to get or to keep something he likes [13] or if he rather gives up the thing he likes in order to avoid the uncomfortable stimulus [14]. With this knowledge, a representative survey question could be: *"Do you think that the animals under your care are able to endure uncomfortable stimuli in order to get something they like?"** (Example: They would be able to choose to endure electric shock or extreme temperatures (cold or hot) in order to eat their favourite food)*.

Providing a definition of play is a challenging task compared to other, more 'serious' behaviours, which are more easily definable [15]. Play can be defined as spontaneous, enjoyable, and voluntary and it occurs when animals are free from environmental and social stress factors [15]. Play requires a higher mental effort than most 'non-aggressive' behaviours because it demands advanced communication skills, improvisation, strategic timing, and creativity [15]. In the light of this information, we can formulate the following representative survey question: *"Do you consider that the animals under your care are capable of playing with other animals (of the same or other species)?"*

Judgement bias refers to the impact of emotions on the way ambiguous information is interpreted [16]. By having animals interpret ambiguous information, judgement bias is used to assess the mood of animals [17]. To do so, animals undergo training to associate a positive stimulus with a reward and another stimulus with the absence of a reward [17]. Then we present them with an ambiguous intermediate stimulus [17]. Animals that respond to the ambiguous stimulus in the same way as the positive stimulus are considered to have a positive, optimistic mood, while those who respond in the same way as the negative stimulus are considered to have a negative, pessimistic mood [17]. Based on this we can formulate the following survey question: *"Do you believe that the mood of animals under your care influences their judgement?"*. (Example: Animals' usual food bowl is placed empty in an unusual location. The animals might or not approach it depending on whether they interpret it as a feeding opportunity or not. Do you believe the animals under your care are more likely to approach it when they are in a good mood, compared to when they are in a bad mood, or experiencing negative emotions?)

Preference testing corresponds to the investigation of animals' preferences regarding various options [18]. Preference testing is used in the study of animal welfare and environmental enrichment [18]. In this test animals are offered several choices, and their preferences are studied [18]. In the light of this knowledge, a representative survey question could be: *"Do you think that animals under your care can have preferences and attribute different values to different items or experiences?"** (Example: Animals under your care are capable of attributing different values to different food items, rewards or enrichments, and will work harder to obtain those they prefer)*

3- Integration at a time

Integration at a time, also called "external synchronic unity", represents an animal's ability to unify their perspective of an experience at any given time [1] and to experience the world from a unique perspective [12]. This dimension of consciousness can be investigated with "interocular transfer", "multi-sensory integration" and "meta-control" [1]. Based on this knowledge, a representative survey question could be: *"Do you believe that the animals under your care perceive the experiences they live in a unifying way?"** (Example: An animal in search of food perceives the steps to get there—its movements, its discretion, its search for food or its hunt—as a continuous action and not as distinct events)*

Interocular transfer is a test that explores the capacity to transfer a learning from one eye to another [19]. In this test animals learn a visual task within the visual field of one of their eyes, and the goal is to study if they are capable of performing the task in the visual field of the other eye [19]. From this a representative survey question could be: *"Do you believe that the animals under your care have the capacity to perform a task in the visual field of one of their eyes when this task has been learned in the visual field of their opposite eye?"*. (Example: We cover the left eye of an animal and teach him to touch a mark with his head. Once he has learned the task, we cover its right eye instead and ask him to touch the mark again. The animal will be able to reproduce the experience with his left eye only)

Multi-sensory integration represents the ability of an animal's brain to integrate information from various senses at the same time [20]. Integration of several senses allows animals to have a better perception of a situation and thus make a decision in favour of their survival [20]. With this information, a representative survey question could be: *"Do you believe that the animals under your care are able to assimilate information that comes from various senses at the same time?"** (Example: They are able to see the movement of a bouncing ball and understand that the sound they hear at the same time is that of the ball. The same would apply for scents, tactile experiences in addition to acoustic and visual stimuli)*

Meta control corresponds to the dominance of one of the two cerebral hemispheres [21], which inhibits the second and is more evident in species with cerebral asymmetry [22]. As each eye communicates with a respective hemisphere, Ünver, Xiao, and Güntürkün proposed an example of meta control in which animals are trained to perform monocular discrimination between a pair of stimuli. Once each hemisphere has learned to discriminate its stimuli, animals are then exposed binocularly to another pair of stimuli composed of the rewarded stimulus from each hemisphere, and the animals' reactions are studied [22]. A question to assess the respondent's perception on this capability in animals could not be formulated.

4 - Integration across time

Integration across time, also referred to as external diachronic unity [1], examines how animals perceive events as a continuous, uninterrupted flow [12]. This dimension can be explored in animals through "apparent motion", "flicker-fusion threshold and considering the presence of a memory of objects and events, sufficient to recognize the identity of individuals or objects and understand the nature of occurring events [1]. Based on this knowledge, a representative survey question could be: *"Do you believe that animals under your care perceive the world and the events in an uninterrupted way?"** (Example: Do you think that the animals under your care perceive the world continuously, without interruption, while reacting to changes in their environment, and not as unrelated momentary events?)*

Apparent motion, also called illusory motion, is a visual illusion in which an animal perceives motion that is not identical to the actual motion present in the physical environment or when the object of their attention is completely static [23]. This phenomenon can be observed with a sequence of stationary lights that are flashed at precise intervals and is useful in order to analyse the mechanisms responsible for the perception of continuous motion in animals [24]. With this knowledge a representative survey question could be: *"Do you believe that animals under your care can experience apparent motion?"* (Example: The same stationary object is presented in sequence to an animal, with different positions and at regular intervals. Despite the stationary nature of each individual frame, the rapid succession creates a visual illusion for the animals under your care, making them perceive the object in continuous motion).

Critical flicker-fusion represents the threshold at which intermittent illumination is perceived as continuous light [25]. This allows the study of temporal resolution in animals [26], which corresponds to the time needed for an animal to acquire data to generate an image [27]. Critical flicker-fusion can be explored with electrophysiological or behavioural methods [26]. Based on this information we can formulate the following survey question: *"Do you consider that the animals under your care can perceive, at a certain frequency, an intermittent illumination as continuous light?".* (Example: Intermittent light is presented to an animal. Gradually, the flicker frequency of this light is increased until reaching a threshold where the animal perceives a steady light instead of a flickering one)

5- Self-consciousness
Self-consciousness is an animal's ability to perceive itself as distinct from its environment and be aware of it [12]. Self-consciousness can be investigated with "bodily/body awareness", "mirror self-recognition" and "mental time travel" [1]. With this understanding, a representative survey question could be: *"Do you think that the animals under your care are aware of their own body position or movement?".*

Bodily/Body self-awareness represents the animal's knowledge of its own body in relation to its environment [28]. It relies on the awareness of position and movement of the body and its parts and the awareness of the body's sensations [29]. This capacity can be evaluated with tests where the animal's own body prevents them from performing a task [28]. In light of this information, we could use the following question as a representative survey question: *"Do you think that the animals under your care are able to understand that their own body prevents them from successfully resolving a problem?"** (Example: they are able to understand that their size prevents them from passing through a narrow path)*

Mirror self-recognition is a well-known method to investigate if an animal can recognize itself [30]. This test is based on the observation of animal's behaviour in reaction to themselves in the mirror with or without a mark [30]. Based on this information we can formulate a representative survey question: *"Do you believe that the animals under your care are able to recognize themselves in a mirror? (as distinct from another individual of the same or other species)"*.*

Mental time travel represents an animal's capacity to remember events in its own past (episodic memory) and to project itself into the future [31]. Investigations of mental time travel are based on the denial of the Bischof-Köhler hypothesis which states that animals are not able to prevent future needs that are different from their actual one's [32]. With this knowledge, a representative survey question

could be: *"Do you think that the animals under your care are able to remember events that happened to them in the past?"**. (Example: They are able to remember the loss of a loved one, or where they stored food)* (In the survey, this question was changed to the section on the "Integration across time" dimension because the specific example used was more relevant to that dimension) or even *"Do you believe that the animals under your care are able to plan their future, to act for their future needs?"**. (Example: They are able to anticipate and set aside food for their future needs)* (In the survey, this question was changed to the section on the "Abstraction" dimension because the specific example used was more relevant to that dimension)

6 - Experience of Agency

The experience of agency refers to whether the animal feels that its actions are voluntarily controlled by itself, rather than just things that happen to it [1]. The authors provide examples of tests used to determine if animals can experience agency such as "response-inhibition" and "delayed gratification tests". Based on this dimension, a representative survey question could be: *"Do you think that the animals under your care are able to understand that they are the one who controls their own actions?"*. (Example: An animal in search of food is aware that the outcome of its search will depend on their ability to control, its actions, discretion, speed, agility, and adaptation to the prey it pursues or the food it seeks to acquire).

Response inhibition is the ability for an animal to restrain his automatic reactions in order to respond to a stimulus more appropriately [33]. This capacity has been tested with the detour reaching tasks [34] and the object displacement tasks [35]. Based on this knowledge, a representative survey question could be: *"Do you think that the animals under your care are able to consciously control their automatic reactions in order to respond more adequately to a stimulus?"*. (Example: An animal suddenly hears a loud and frightening noise, but can consciously control its automatic reaction to flee or attack in order to respond more appropriately and ensure its safety)

Detour reaching tasks evaluate the capacity for an animal to develop indirect strategies to reach a reward when reaching directly the reward is not possible [34]. In this task animals must make a detour around a transparent barrier in order to get the reward [36]. The visual information of the reward right in front of the subject contradicts the tactile information suggesting the presence of a barrier [36]. Variations of the initial test can be conducted, as seen in the article by Vlamings, Hare and Call (2010), where the animal has to reach around a barrier to access the reward. In the light of this information, we can formulate the following representative survey question: *"Do you think that the animals under your care are able to understand when they cannot directly achieve a reward and therefore implement indirect strategies to achieve it?"** (Example: Animals are placed in front of a transparent box with food inside that they cannot reach directly. On the side of this box there is a small trap that they can open to reach the food. Animals are able to understand it and reach the food through this trap)*

The ability essential for an animal to track objects, to remember where food is located, is investigated with the object displacement task [35]. In this task, an animal must find an object hidden under a cup, which is then moved among other cups [35]. Animal's ability to understand that the object continues to exist even when it is not visible, and to remember the location of the object, is evaluated [35]. Based

on this information, a representative survey question could be: *"Do you believe that the animals under your care are able to remember the localisation of objects?"*. (Example: According to you, do the animals under your care can remember that they found food in a specific location and return to it?)

Delayed gratification is the ability for an animal to forgo an immediate reward in order to access a better one in the long term [37]. Binary choice tests are a way to test animals ability to delay gratification by presenting them with two choices, they can either take the reward now or wait [38]. Binary choice tests can be realised by presenting food [39] or comestible tools that can be used to get a more preferred food [40]. Some of the binary choice tests allowed animals to modify their choice once made, in order to analyse commitment to the initial choices [41]. But in most of these tests, once the animal makes a choice he can no longer change it [41]. With this concept, a representative survey question could be: *"Do you think that the animals under your care are able to forgo an immediate reward in order to obtain a better one in the long term?"**. (Example: We present a piece of food to an animal but hold a favourite food item in our hand. It can either take the food or wait to receive the preferred food later. The animal is capable of understanding and waiting for the preferred food)*

Delay maintenance tasks enable the study of the degree of self-control. They analyse at which point the animal is no longer able to control himself [41]. There are different types of delay maintenance tasks, either the addition of rewards one by one until the animal chooses to obtain the accumulated reward, or the reward is permanently available and the animal has to control himself continuously to get the better one [41]. With this information, a representative survey question would be: *"Do you believe that the animals under your care can learn to control themselves and their impulses, even in front of a significant amount of food, in order to get a larger or a better reward ?"*. (Example: An animal has in front of him, at his disposal, a piece of food that he prefers. In front of him is placed a bowl filled with this same food. A keeper gives the animal food one by one until the animal takes the food but stops adding food once the animal eats. The animals under your care are able to control their desire to eat the available food because they understand that they can get all the food in the bowl).

7 - Experience of Ownership

The experience of ownership represents the capacity for an animal to perceive its body parts as belonging to it [1]. "Body-world discrimination" and "rubber-hand or rubber-tail illusions" are ways to test this capacity [1]. According to this we can formulate the following survey question: *"Do you believe that the animals under your care are able to perceive their body parts as their own?"**.

No article was found including the definition of the term Body-world discrimination.

Rubber-hand and rubber-tail illusions are experiments that test the ability for an animal to perceive body-parts as its own [42-43]. In these experiments, the hand or the tail of the animal is hidden and replaced by a fake one visible to the animal [42]. Both are touched simultaneously, or separately and the animal response is measured [42]. Based on these experiments, a representative survey question could be: *"Do you believe that the animals under your care are able to consider a rubber-hand or rubber-tail as their own hand or tail ?"** (Example: The animals will recognize or react to a replica of one of their body parts (placed in proximity to the real part) as if it were their own)*

8 - Reasoning

Reasoning represents the ability to form thoughts and develop reasoning in different areas [1]. This dimension can be investigated through "transitive inference", "mindreading", "metacognition" and "tool-use" [1]. Based on this dimension of consciousness, a representative survey question could be: *"Do you think that the animals under your care can have developed thoughts and reason on a subject?"*.

Transitive inference is a form of reasoning where an animal, from initial information, manages to deduce a logical conclusion [44]. It may be investigated with the "n-term" series task [44]. From this knowledge, we can formulate a representative survey question: *"Do you think that the animals under your care are capable of inferring logical conclusions from initial information?"**. (Example: When an animal notices the surroundings getting darker (initial information), it will seek shelter to protect itself from a possible danger)*

The "n-term" series task is a method initially used for the study of children that has been adapted to the study of animals [44]. In this task, a precise number of stimuli (n) is presented to an animal. Animals have reinforcement information only between adjacent stimuli (A and B or B and C) and their ability to infer results between non-adjacent stimuli (A and C) is evaluated [44]. With this knowledge a representative survey question could be: *"Do you think that animals under your care can anticipate outcomes between two non-adjacent stimuli using results from comparisons between adjacent stimuli?"*. (Example: An animal is presented with images of fruits, some of which are associated with a reward. The animal learns that if it chooses the banana image (A) over the apple image (B), it will be rewarded. Similarly, if it chooses the apple image (B) over the orange image (C), it will be rewarded. If the banana image (A) and the orange image (C) are then presented, the animal will choose the banana image (A) through deduction)

Mindreading, also known as Theory of Mind [45] is the capacity for an animal to infer other's thoughts, mental states, desires, beliefs and even goals [46]. It is used as a criterion to assess an evolved social cognition similar to that of humans and serves as a marker of cognitive sophistication in the realm of social interactions [47]. This capacity allows animals to communicate, to understand, interpret or predict other's actions [46]. This dimension can be explored through experiments such as the "desire-understanding sharing experiment", and the false belief experiments [46]. Based on this information, we can formulate the following representative survey question: *"Do you think that the animals under your care are capable of consciously deducing the thoughts, mental states, desires, beliefs and even the goals of others ?"** (Example: Two animals face each other separated by a barrier. Only one of them receives food. The one who has received food is able to deduce that the other's desire to eat and hopes to receive food also)*

The desire-understanding sharing experiment is a method that studies the capacity for an animal to understand and respond to another's desire [46]. To do this, a specific food desire is induced in one animal, and the reaction of an adjacent animal that possesses this food is [46]. From this experiment, a representative survey question could be: *"Do you believe that the animals under your care are able to deduce another's desires and help satisfy them?"* (Example: Two animals face each other, separated by a barrier. Only one of them receives food. The one who has received food deduces the other's desire to receive

the same food and shares it with him) (or even "Do you think that the animals under your care can understand and share the desired food with another?" *(Example: Two animals face each other, separated by a barrier. Only one of them receives food. The one who has received food deduces the other's desire to receive the same food and shares it with him. Even if he desires this food too)*, "Do you believe that the animals under your care are able to voluntarily share their food to meet the desire of another?" *(Example: Two animals face each other, separated by a barrier. Only one of them receives food. The one who has received food deduces the other's desire to receive the same food and shares it with him)*.

The false belief experiment is a standard test to assess "Theory of Mind", but while numerous studies have been conducted in humans, there have been few studies conducted in animals [48]. Understanding false beliefs means recognizing that the actions of others are influenced not by the actual state of things but by their beliefs about reality. It involves an awareness that these beliefs might be different from one's own [47]. This experiment involves placing an animal in a situation where another animal has a false belief about the location or the nature of an object, to see if the animal reacts, taking into account that incorrect belief [47]. In the light of this information, a representative survey question could be: *"Do you believe that the animals under your care are able to recognize that the actions of others are influenced by their beliefs?"**. (Example: An animal is presented with an object that is then hidden under a cup in its presence. When the animal is absent, the object is moved under another cup. Another animal observes the scene. He knows the new location of the object and instinctively looks at the initial cup when the first animal returns. He anticipates its counterpart's false belief, knowing that the first animal thinks the object is still at its initial position)*

Metacognition involves the awareness and comprehension of an animal's own mental process and cognitive states [49]. In order to test whether an animal exhibits metacognition, it is subjected to a duration-discrimination test [49]. The animal has the option to either perform or refuse the test [49]. Refusing the test is associated with a small reward, succeeding in the test is associated with a large reward, while failure is associated with no reward [49]. If the animal possesses metacognition, it will more often refuse to take the test when the difficulty is high [49]. Regarding this information, a representative survey question could be: *"Do you believe that the animals under your care are aware of their own mental process and cognitive states?"* (Example: The animal can recognize that a test is too difficult for him and, therefore, chooses not to respond to it)

Tool-use involves the act of modifying the shape, position, or state of another object, organism, or the user themselves while holding or carrying the tool during or just before its use [50]. Long considered as unique to the human species, the ability to use tools has been demonstrated in certain animals such as elephants, dolphins, chimpanzees, as well as in some bird species [51]. The question of whether animals use tools for a specific purpose has been raised and explored in certain animal species where investigation was possible [50]. It has been found that in some species (especially chimpanzees, capuchin monkeys, and New Caledonian crows) tool use is guided for specific purposes [50]. In light of this information, a representative survey question could be: *"Do you believe that the animals under your care are able to use tools for a specific purpose?"** (Example: Do you believe that the animals under your care are able to use a tool in order to get food?)*

9 - Learning

Animals' ability to learn things can be investigated with "trace conditioning", "reversal learning", "imitation learning" and "one trial learning" [1]. From this dimension, a representative survey question could be: *"Do you think that the animals under your care are able to learn things or learn to do things?"**

Trace conditioning is a way to study animals' capacity to learn to associate events separated in the time [52]. This associative learning can be evaluated through auditory, taste, visual and olfactory systems [52]. With this information a representative survey question could be: *"Do you think that the animals under your care are able to learn to associate two distant events in time?"** (Example: They associate the sound of a bell with the delivery of food some time later)*

Reversal learning represents the ability for an animal to learn to make a discrimination between two stimuli and then to reverse his choice when both stimuli are redefined [53]. To do so, the animal has to learn to respond to a positive stimulus [53]. Once the animal shows a high success rate of response, the stimulus is redefined: the positive stimulus becomes the negative one, and vice versa. If the animal reverses his choice, it indicates that he has 'learned to learn'. With this knowledge, a representative survey question could be: *"Do you think that the animals under your care are able to learn to discriminate between two choices and then reverse their choice when the two stimuli are redefined?"*. (Example: Two different images are presented to an animal. When the animal chooses the image with a tree, he receives a reward (positive stimulus), and when he chooses the image with a sun, he receives nothing. When the animal has a high success rate in choosing the image with a tree, the image associated with the reward becomes the one with a sun. The animal adapts, changes his choice, and selects the image with the sun)

Imitation learning represents animals' ability to learn to reproduce the action of someone else [54]. In some cases, animal imitation is adaptive [55]. By copying the current behaviour of other group members, individuals of social species can benefit from the knowledge of others and escape predators [55]. Behavioural synchrony, goal emulation, social mimicry, and contextual imitation are examples of imitation learning [55]. Based on this knowledge, we can formulate the following representative survey question: *"Do you believe that the animals under your care are able to reproduce the actions of another animal by imitation?"**

Behavioural synchrony, also known as contagion, refers to the phenomenon where animals copy the behaviour of other group members in a synchronised way [55]. Behavioural synchronisation requires that the animal recognize the action of others; Group grooming or coordinated movements during bird flights are examples of behavioural synchronisation [55]. In the light of this knowledge, we can formulate the following survey question: *"Do you think that animals under your care are able to copy the behaviour of other group members synchronously?"*. (Example: The animals under your care can join others and imitating their behaviour at the same time for hunting or moving in a coordinated manner)

Goal emulation represents the fact that an animal is influenced after observing another animal achieve a goal and receive a reward [55]. Goal emulation involves an understanding of the motivations and objectives of others [55]. From this information a representative survey question could be: *"Do you believe that the animals under your care, after observing others achieve a goal and receive a reward, are influenced and attempt to achieve the same reward?"** (Example: An animal observes one of its conspecifics

successfully obtaining food using a particular technique. The animal is then influenced and attempts to reproduce the same technique to achieve the same goal)*

Social mimicry consists of animals imitating the facial expressions, postures or manners of other animals in order to communicate a social message with them [55]. Contagious yawning and greeting are examples of this type of imitation [55]. Considering this information a representative survey question could be: *"Do you believe that the animals under your care are capable of imitating the facial expressions, postures or manners of other animals to communicate a social message?".* (Example: When animals exhibit specifics sounds, gestures or body positions, the animals under your care will imitate the same patterns to convey a social message)

Contextual imitation refers to the imitation of actions or behaviours, already known to the animal that imitates the other, applied to a new context [55]. In the light of this information a representative survey question could be: *"Do you believe that the animals under your care can mimic actions they are already familiar with, but in a context different from their usual?"**. (Example: The animals under your care have developed specific food-seeking behaviours. If placed in a different environment, they will replicate these same behaviours)*

10 - Abstraction

Abstraction is the act of focusing on a specific characteristic of something, disregarding the rest, and applying this focus to everything that shares that same characteristic [56]. This dimension can be investigated with "conceptual categorization" and "perspective-taking" [1]. Based on this dimension we can formulate the following representative survey question: *"Do you think that the animals under your care are able to focus on something specific disregarding the rest and apply this to over elements that share this same characteristic?"**. (Example: The animals under your care encounter shiny and matte objects in their environment. When they retrieve shiny objects, they receive a reward. Subsequently, they develop an abstraction by focusing solely on shiny objects)*

Conceptual categorisation refers to animals' capacity to recognise and categorise elements into categories [57]. Unlike perceptual categorisation, conceptual categorisation is based on an understanding of the nature and functions of objects, as well as their roles in various events [3]. In the light of this information, a representative survey question could be: *"Do you consider that the animals under your care are able to categorise elements into categories according to their nature, functions and roles?"**. (Example: Animals under your care recognise and categorise a wooden stick and a stone in the category: "tools used to get food")*

Perspective-taking represents the ability for an animal to see the world from another animal's point of view [58]. It relies on communication, memory, language, and perception and plays a fundamental role in understanding social dynamics across various situations [58]. It is a useful way for animals to infer what other animals know and anticipate what they will do next [59]. From this information, a representative survey question could be: *"Do you conceive that the animals under your care are able to see the world from another animal's point of view?"**.

(* Questions and examples chosen for the survey)

References:

1. Dung, L.; Newen, A. Profiles of Animal Consciousness: A Species-Sensitive, Two-Tier Account to Quality and Distribution. *Cognition* **2023**, *235*, 105409. https://doi.org/10.1016/j.cognition.2023.105409.
2. Güntürkün, O.; Koenen, C.; Iovine, F.; Garland, A.; Pusch, R. The Neuroscience of Perceptual Categorization in Pigeons: A Mechanistic Hypothesis. *Learn Behav* **2018**, *46* (3), 229–241. https://doi.org/10.3758/s13420-018-0321-6.
3. Arterberry, M. E.; Bornstein, M. H. Infant Perceptual and Conceptual Categorization: The Roles of Static and Dynamic Stimulus Attributes. *Cognition* **2002**, *86* (1), 1–24. https://doi.org/10.1016/S0010-0277(02)00108-7.
4. Zhang, J.; Wermter, S.; Sun, F.; Zhang, C.; Engel, A. K.; Röder, B.; Fu, X.; Xue, G. Editorial: Cross-Modal Learning: Adaptivity, Prediction and Interaction. *Front Neurorobot* **2022**, *16*, 889911. https://doi.org/10.3389/fnbot.2022.889911.
5. Zhang, L.-Z.; Zhang, S.-W.; Wang, Z.-L.; Yan, W.-Y.; Zeng, Z.-J. Cross-Modal Interaction between Visual and Olfactory Learning in Apis Cerana. *J Comp Physiol A* **2014**, *200* (10), 899–909. https://doi.org/10.1007/s00359-014-0934-y.
6. Laska, M. Olfactory Discrimination Learning in an Outbred and an Inbred Strain of Mice. *CHEMSE* **2015**, *40* (7), 489–496. https://doi.org/10.1093/chemse/bjv032.
7. Kurt, S.; Ehret, G. Auditory Discrimination Learning and Knowledge Transfer in Mice Depends on Task Difficulty. *Proc. Natl. Acad. Sci. U.S.A.* **2010**, *107* (18), 8481–8485. https://doi.org/10.1073/pnas.0912357107.
8. Treviño, M.; Oviedo, T.; Jendritza, P.; Li, S.-B.; Köhr, G.; De Marco, R. J. Controlled Variations in Stimulus Similarity during Learning Determine Visual Discrimination Capacity in Freely Moving Mice. *Sci Rep* **2013**, *3* (1), 1048. https://doi.org/10.1038/srep01048.
9. Meagher, R. K.; Strazhnik, E.; Von Keyserlingk, M. A. G.; Weary, D. M. Assessing the Motivation to Learn in Cattle. *Sci Rep* **2020**, *10* (1), 6847. https://doi.org/10.1038/s41598-020-63848-1.
10. Schumacher, J. W.; McCann, M. K.; Maximov, K. J.; Fitzpatrick, D. Selective Enhancement of Neural Coding in V1 Underlies Fine-Discrimination Learning in Tree Shrew. *Current Biology* **2022**, *32* (15), 3245-3260.e5. https://doi.org/10.1016/j.cub.2022.06.009.
11. Cohen, Y.; Putrino, D.; Wilson, D. A. Dynamic Cortical Lateralization during Olfactory Discrimination Learning. *The Journal of Physiology* **2015**, *593* (7), 1701–1714. https://doi.org/10.1113/jphysiol.2014.288381.
12. Birch, J.; Schnell, A. K.; Clayton, N. S. Dimensions of Animal Consciousness. *Trends in Cognitive Sciences* **2020**, *24* (10), 789–801. https://doi.org/10.1016/j.tics.2020.07.007.
13. Gibbons, M.; Versace, E.; Crump, A.; Baran, B.; Chittka, L. Motivational Trade-Offs and Modulation of Nociception in Bumblebees. *Proc. Natl. Acad. Sci. U.S.A.* **2022**, *119* (31), e2205821119. https://doi.org/10.1073/pnas.2205821119.
14. Adcock, S. J. J.; Tucker, C. B. Injury Alters Motivational Trade-Offs in Calves during the Healing Period. *Sci Rep* **2021**, *11* (1), 6888. https://doi.org/10.1038/s41598-021-86313-z.
15. Palagi, E.; Burghardt, G. M.; Smuts, B.; Cordoni, G.; Dall'Olio, S.; Fouts, H. N.; Řeháková-Petrů, M.; Siviy, S. M.; Pellis, S. M. Rough-and-tumble Play as a Window on Animal Communication. *Biological Reviews* **2016**, *91* (2), 311–327. https://doi.org/10.1111/brv.12172.
16. Roelofs, S.; Boleij, H.; Nordquist, R. E.; Van Der Staay, F. J. Making Decisions under Ambiguity: Judgment Bias Tasks for Assessing Emotional State in Animals. *Front. Behav. Neurosci.* **2016**, *10*. https://doi.org/10.3389/fnbeh.2016.00119.
17. Bateson, M.; Nettle, D. Development of a Cognitive Bias Methodology for Measuring Low Mood in Chimpanzees. *PeerJ* **2015**, *3*, e998. https://doi.org/10.7717/peerj.998.
18. DePasquale, C.; Sturgill, J.; Braithwaite, V. A. A Standardized Protocol for Preference Testing to Assess Fish Welfare. *JoVE* **2020**, No. 156, 60674. https://doi.org/10.3791/60674.

19. Ortega, L. J.; Stoppa, K.; Güntürkün, O.; Troje, N. F. Limits of Intraocular and Interocular Transfer in Pigeons. *Behavioural Brain Research* **2008**, *193* (1), 69–78. https://doi.org/10.1016/j.bbr.2008.04.022.
20. Stein, B. E.; Stanford, T. R.; Rowland, B. A. Development of Multisensory Integration from the Perspective of the Individual Neuron. *Nat Rev Neurosci* **2014**, *15* (8), 520–535. https://doi.org/10.1038/nrn3742.
21. Kavcic, V.; Fei, R.; Hu, S.; Doty, R. W. Hemispheric Interaction, Metacontrol, and Mnemonic Processing in Split-Brain Macaques. *Behavioural Brain Research* **2000**, *111* (1–2), 71–82. https://doi.org/10.1016/S0166-4328(00)00141-8.
22. Ünver, E.; Xiao, Q.; Güntürkün, O. Meta-Control in Pigeons (Columba Livia) and the Role of the Commissura Anterior. *Symmetry* **2019**, *11* (2), 124. https://doi.org/10.3390/sym11020124.
23. Gori, S.; Agrillo, C.; Dadda, M.; Bisazza, A. Do Fish Perceive Illusory Motion? *Sci Rep* **2014**, *4* (1), 6443. https://doi.org/10.1038/srep06443.
24. Newsome, W. T.; Mikami, A.; Wurtz, R. H. Motion Selectivity in Macaque Visual Cortex. III. Psychophysics and Physiology of Apparent Motion. *Journal of Neurophysiology* **1986**, *55* (6), 1340–1351. https://doi.org/10.1152/jn.1986.55.6.1340.
25. Landis, C.; Hamwi, V. *The Effect of Certain Physiological Determinants on the Flicker-Fusion Threshold*. *Journal of Applied Physiology* **1954**, *6* (9), 566–572. https://doi.org/10.1152/jappl.1954.6.9.566.
26. Lisney, T. J.; Ekesten, B.; Tauson, R.; Håstad, O.; Ödeen, A. Using Electroretinograms to Assess Flicker Fusion Frequency in Domestic Hens Gallus Gallus Domesticus. *Vision Research* **2012**, *62*, 125–133. https://doi.org/10.1016/j.visres.2012.04.002.
27. Benjamin, M. M.; Shaker, M.; Rabbat, M. G. Chapter 5 - Assessing Coronary Artery Disease Using Coronary Computed Tomography Angiography. In *Cardiovascular and Coronary Artery Imaging*; El-Baz, A. S., Suri, J. S., Eds.; Academic Press, 2022; pp 129–145. https://doi.org/10.1016/B978-0-12-822706-0.00011-1.
28. Dale, R.; Plotnik, J. M. Elephants Know When Their Bodies Are Obstacles to Success in a Novel Transfer Task. *Sci Rep* **2017**, *7* (1), 46309. https://doi.org/10.1038/srep46309.
29. DeGrazia, D. Self-Awareness in Animals. In *The Philosophy of Animal Minds*; Lurz, R. W., Ed.; Cambridge University Press, 2009; pp 201–217. https://doi.org/10.1017/CBO9780511819001.012.
30. Gallup, G. G. Chimpanzees: Self-Recognition. *Science* **1970**, *167* (3914), 86–87. https://doi.org/10.1126/science.167.3914.86.
31. Suddendorf, T.; Corballis, M. C. The Evolution of Foresight: What Is Mental Time Travel, and Is It Unique to Humans? *Behav Brain Sci* **2007**, *30* (3), 299–313. https://doi.org/10.1017/S0140525X07001975.
32. Cheke, L. G.; Clayton, N. S. Eurasian Jays (Garrulus Glandarius) Overcome Their Current Desires to Anticipate Two Distinct Future Needs and Plan for Them Appropriately. *Biol Lett* **2012**, *8* (2), 171–175. https://doi.org/10.1098/rsbl.2011.0909.
33. Szabo, B.; Noble, D. W. A.; Whiting, M. J. Context-Specific Response Inhibition and Differential Impact of a Learning Bias in a Lizard. *Anim Cogn* **2019**, *22* (3), 317–329. https://doi.org/10.1007/s10071-019-01245-6.
34. Vlamings, P. H. J. M.; Hare, B.; Call, J. Reaching around Barriers: The Performance of the Great Apes and 3–5-Year-Old Children. *Anim Cogn* **2010**, *13* (2), 273–285. https://doi.org/10.1007/s10071-009-0265-5.
35. Barth, J.; Call, J. Tracking the Displacement of Objects: A Series of Tasks with Great Apes (Pan Troglodytes, Pan Paniscus, Gorilla Gorilla, and Pongo Pygmaeus) and Young Children (Homo Sapiens). *Journal of Experimental Psychology: Animal Behavior Processes* **2006**, *32* (3), 239–252. https://doi.org/10.1037/0097-7403.32.3.239.
36. Taylor, J. R.; Elsworth, J. D.; Roth, R. H.; Sladek, J. R.; Redmond, D. E. COGNITIVE AND MOTOR DEFICITS IN THE ACQUISITION OF AN OBJECT RETRIEVAL/DETOUR TASK IN MPTP-TREATED MONKEYS. *Brain* **1990**, *113* (3), 617–637. https://doi.org/10.1093/brain/113.3.617.

37. Beran, M. J. Maintenance of Self-Imposed Delay of Gratification by Four Chimpanzees (*Pan Troglodytes*) and an Orangutan (*Pongo Pygmaeus*). *The Journal of General Psychology* **2002**, *129* (1), 49–66. https://doi.org/10.1080/00221300209602032.
38. Anderson, J. R.; Kuroshima, H.; Fujita, K. Delay of Gratification in Capuchin Monkeys (Cebus Apella) and Squirrel Monkeys (Saimiri Sciureus). *Journal of Comparative Psychology* **2010**, *124* (2), 205–210. https://doi.org/10.1037/a0018240.
39. Stevens, J. R.; Hallinan, E. V.; Hauser, M. D. The Ecology and Evolution of Patience in Two New World Monkeys. *Biol Lett* **2005**, *1* (2), 223–226. https://doi.org/10.1098/rsbl.2004.0285.
40. Evans, T. A.; Westergaard, G. C. Self-Control and Tool Use in Tufted Capuchin Monkeys (Cebus Apella). *Journal of Comparative Psychology* **2006**, *120* (2), 163–166. https://doi.org/10.1037/0735-7036.120.2.163.
41. Evans, T. A.; Beran, M. J. Delay of Gratification and Delay Maintenance by Rhesus Macaques (Macaca Mulatta). *The Journal of General Psychology* **2007**, *134* (2), 199–216. https://doi.org/10.3200/GENP.134.2.199-216.
42. Shokur, S.; O'Doherty, J. E.; Winans, J. A.; Bleuler, H.; Lebedev, M. A.; Nicolelis, M. A. L. Expanding the Primate Body Schema in Sensorimotor Cortex by Virtual Touches of an Avatar. *Proc. Natl. Acad. Sci. U.S.A.* **2013**, *110* (37), 15121–15126. https://doi.org/10.1073/pnas.1308459110.
43. Wada, M.; Takano, K.; Ora, H.; Ide, M.; Kansaku, K. The Rubber Tail Illusion as Evidence of Body Ownership in Mice. *J. Neurosci.* **2016**, *36* (43), 11133–11137. https://doi.org/10.1523/JNEUROSCI.3006-15.2016.
44. Vasconcelos, M. Transitive Inference in Non-Human Animals: An Empirical and Theoretical Analysis. *Behavioural Processes* **2008**, *78* (3), 313–334. https://doi.org/10.1016/j.beproc.2008.02.017.
45. Hill, H.; Dietrich, S.; Cadena, A.; Raymond, J.; Cheves, K. More than a Fluke: Lessons Learned from a Failure to Replicate the False Belief Task in Dolphins. *IJCP* **2018**, *31*. https://doi.org/10.46867/ijcp.2018.31.01.13.
46. Krupenye, C.; Call, J. Theory of Mind in Animals: Current and Future Directions. *WIRES Cognitive Science* **2019**, *10* (6), e1503. https://doi.org/10.1002/wcs.1503.
47. Kano, F.; Krupenye, C.; Hirata, S.; Tomonaga, M.; Call, J. Great Apes Use Self-Experience to Anticipate an Agent's Action in a False-Belief Test. *Proc. Natl. Acad. Sci. U.S.A.* **2019**, *116* (42), 20904–20909. https://doi.org/10.1073/pnas.1910095116.
48. O'Connell, S.; Dunbar, R. I. M. A Test for Comprehension of False Belief in Chimpanzees. **2003**.
49. Foote, A. L.; Crystal, J. D. Metacognition in the Rat. *Current Biology* **2007**, *17* (6), 551–555. https://doi.org/10.1016/j.cub.2007.01.061.
50. Seed, A.; Byrne, R. Animal Tool-Use. *Current Biology* **2010**, *20* (23), R1032–R1039. https://doi.org/10.1016/j.cub.2010.09.042.
51. Striedter, G. F. Bird Brains and Tool Use: Beyond Instrumental Conditioning. *Brain Behav Evol* **2013**, *82* (1), 55–67. https://doi.org/10.1159/000352003.
52. Moye, T. B.; Rudy, J. W. Ontogenesis of Trace Conditioning in Young Rats: Dissociation of Associative and Memory Processes. *Dev. Psychobiol.* **1987**, *20* (4), 405–414. https://doi.org/10.1002/dev.420200405.
53. Bublitz, A.; Weinhold, S. R.; Strobel, S.; Dehnhardt, G.; Hanke, F. D. Reconsideration of Serial Visual Reversal Learning in Octopus (Octopus Vulgaris) from a Methodological Perspective. *Frontiers in Physiology* **2017**, *8*.
54. Buttelmann, D.; Carpenter, M.; Call, J.; Tomasello, M. Enculturated Chimpanzees Imitate Rationally. *Developmental Sci* **2007**, *10* (4), F31–F38. https://doi.org/10.1111/j.1467-7687.2007.00630.x.
55. Bates, L. A.; Byrne, R. W. Imitation: What Animal Imitation Tells Us about Animal Cognition. *WIRES Cognitive Science* **2010**, *1* (5), 685–695. https://doi.org/10.1002/wcs.77.
56. Galizio, M.; Bruce, K. E. Abstraction, Multiple Exemplar Training and the Search for Derived Stimulus Relations in Animals. *Perspect Behav Sci* **2018**, *41* (1), 45–67. https://doi.org/10.1007/s40614-017-0112-y.

57. Martin-Malivel, J.; Fagot, J. Cross-Modal Integration and Conceptual Categorization in Baboons. *Behavioural Brain Research* **2001**, *122* (2), 209–213. https://doi.org/10.1016/S0166-4328(01)00179-6.
58. Labash, A.; Aru, J.; Matiisen, T.; Tampuu, A.; Vicente, R. Perspective Taking in Deep Reinforcement Learning Agents. *Front. Comput. Neurosci.* **2020**, *14*, 69. https://doi.org/10.3389/fncom.2020.00069.
59. Huber, L.; Lonardo, L. Canine Perspective-Taking. *Anim Cogn* **2023**, *26* (1), 275–298. https://doi.org/10.1007/s10071-022-01736-z.
