# Supplementary material for: A Global Survey on the Perception of Conservationists Regarding Animal Consciousness
Source: Animals (Basel). 2025 Jan 24;15(3):341. doi: 10.3390/ani15030341 (PMC11816229; doi:10.3390/ani15030341)
Supplement: Supplementary file 1 [file animals-15-00341-s001.zip › File S2.pdf]

# **File S2.** Full Questionnaire

## Perception of caretakers on animal consciousness in wildlife conservation

I would like to invite you to share your views on animal consciousness. This survey is part of my final Thesis in Veterinary Medicine at Escola Universitária Vasco da Gama (EUVG), Coimbra, Portugal. The study aims to assess the perceptions of caretakers in conservation centres regarding the level of consciousness of the animals under their care.

Your participation in this survey is confidential. I ensure the security of your information by maintaining anonymity at all stages of the study, from data collection to analysis, and publication of the results. You will be asked to answer some questions about yourself, and then about the species you work with. The survey should take less than ten minutes to complete, and participation is voluntary.

No perceived risks in taking part in this survey were identified and you may abandon the survey at any time. By submitting your response, you will help advance our current knowledge of animal consciousness. This study has been approved by the Ethics Committee of the EUVG.

If you have any questions, please contact me at valentine.yakhlef3@gmail.com. Your experiences and perspectives are invaluable, and I am deeply grateful for your time and input.

Sincerely,

Valentine Yakhlef

I confirm that I have read and am aware of: (i) the study aims and methods (ii) the contact of the person responsible for further inquiries, and (iii) the option to abandon the survey before submission. Additionally, I consent to the anonymous storage and utilization of the data provided exclusively for the present purposes. *

Yes
No

## Demographic information

What is your nationality? *

What is your country of residence? *

How old are you? *

< 18
18 - 24
25 - 34
35 - 44
45 - 54
55 - 64
65 - 74
75 or more

What is your gender? *

Female
Male
Other:

What is your level of education? *

None
Primary education
Lower secondary education
Upper secondary education
Bachelor's or equivalent
Master's or equivalent
Doctorate or equivalent

Which of these options best describes your profession? *

Animal keeper
Veterinarian
Ecologist
Biologist
Student or Intern
Other:

**Please write the common or scientific name of a single species you work with.** Please consider the species you know best and focus only on this species for the rest of the survey.

For how long have you worked with this species (in years)? *

## Perceptual-Richness

*Please indicate your level of agreement with the following statements about the animals of the species that you work with. (please consider only the single species named above)*

**They can recognize elements in their surroundings and categorise them**

*(Example: They see a keeper and are able to classify him into the category "Human", or they see an insect, a fruit, a bottle of milk or a meat, and classify it into the category "Food")*

1 (Totally disagree) 2 3 4 5 (Totally Agree)

**The animals of this species are able to react differently to a stimulus, depending on whether it's a rewarded stimulus or an unrewarded one**

*(Example: Animals might react differently to a husbandry cue depending on whether or not you are holding a food reward item)*

1 (Totally disagree) 2 3 4 5 (Totally Agree)

## Evaluative-Richness

*Please indicate your level of agreement with the following statements about the animals of this species:*

**They can perceive and evaluate their own internal or external environments, and respond accordingly**

*(Example: They perceive the change in temperature when they arrive in a cold or hot place and they perceive the change in their emotions when they move from well-being to sadness)*

1 (Totally disagree) 2 3 4 5 (Totally Agree)

**They are able to endure uncomfortable stimuli in order to get something they like**

*(Example: They would be able to chose to endure electric shock or extreme temperature (very cold or very hot) in order to eat their favourite food)*

1 (Totally disagree) 2 3 4 5 (Totally Agree)

**They can have preferences and attribute different values to different items or experiences**
*(Example: The animals under your care are capable of attributing different values to different food items, rewards or enrichments, and will work harder to obtain those they prefer)*

1 (Totally disagree) 2 3 4 5 (Totally Agree)

## Integration at a time

*Please indicate your level of agreement with the following statements about the animals of this species:*

**They perceive the experiences they live in a unifying way**

*(Example: An animal in search of food perceives the steps to get there—its movements, its discretion, its search for food or its hunt—as a continuous action and not as distinct events)*

1 (Totally disagree) 2 3 4 5 (Totally Agree)

**They are able to assimilate information that comes from various senses at the same time**

*(Example: They are able to see the movement of a bouncing ball and understand that the sound they hear at the same time is that of the ball. The same would apply for scents, tactile experiences in addition to acoustic and visual stimuli)*

1 (Totally disagree) 2 3 4 5 (Totally Agree)

## Integration across time

*Please indicate your level of agreement with the following statements about the animals of this species:*

**They are able to remember events that happened to them in the past**

*(Example: They are able to remember the loss of a loved one, or where they stored food)*

1 (Totally disagree) 2 3 4 5 (Totally Agree)

**They perceive the world and the events in an uninterrupted way**

*(Example: Do you think that the animals under your care perceive the world continuously, without interruption, while reacting to changes in their environment, and not as unrelated momentary events?)*
1 (Totally disagree) 2 3 4 5 (Totally Agree)

## Self-consciousness

*Please indicate your level of agreement with the following statements about the animals of this species:*

**They are able to understand that their own body prevents them from successfully resolving a problem**

*(Example: They are able to understand that their size prevents them from passing through a narrow path)*

1 (Totally disagree) 2 3 4 5 (Totally Agree)

**They are able to recognize themselves in a mirror (as distinct from another individual of the same or other species)**

1 (Totally disagree) 2 3 4 5 (Totally Agree)

## Experience of Agency

*Please indicate your level of agreement with the following statements about the animals of this species:*

**They are able to understand when they cannot directly achieve a reward and therefore implement indirect strategies to achieve it**

*(Example: Animals are placed in front of a transparent box with food inside that they cannot reach directly. On the side of this box there is a small trap that they can open to reach the food. Animals are able to understand it and reach the food through this trap)*

1 (Totally disagree) 2 3 4 5 (Totally Agree)

**They are able to forgo an immediate reward in order to obtain a better one in the long term**

*(Example: You present a piece of food to an animal but show a favourite food item in your hand. It can either take the food or wait in order to receive the preferred food later. The animal is capable of understanding and waiting for the preferred food)*

1 (Totally disagree) 2 3 4 5 (Totally Agree)

## Experience of Ownership

Please indicate your level of agreement with the following statements about the animals of this species:

**They perceive their body parts as their own.**

1 (Totally disagree) 2 3 4 5 (Totally Agree)

**They consider a rubber-hand or rubber-tail as their own hand or tail**

*(Example: The animals will recognize or react to a replica of one of their body parts (placed in proximity to the real part) as if it were their own)*

1 (Totally disagree) 2 3 4 5 (Totally Agree)

## Reasoning

Please indicate your level of agreement with the following statements about the animals of this species:

**They are capable of inferring logical conclusions from initial information**

*(Example: When an animal notices the surroundings getting darker (initial information), it will seek shelter to protect itself from a possible danger)*

1 (Totally disagree) 2 3 4 5 (Totally Agree)

**They are capable of consciously deducing the thoughts, mental states, desires, beliefs and even the goals of others.**

*(Example: Two animals face each other separated by a barrier. Only one of them receives food. The one who has received food is able to deduce that the other desires to eat and hopes to receive food also)*

1 (Totally disagree) 2 3 4 5 (Totally Agree)

**They are able to recognize that the actions of others are influenced by their beliefs**
*(Example: An animal is presented with an object that is then hidden under a cup in its presence. When the animal is absent, the object is moved under another cup. Another animal observes the scene. He knows the new location of the object and instinctively looks at the initial cup when the first animal returns. He anticipates its counterpart's false belief, knowing that the first animal thinks the object is still at its initial position)*

1 (Totally disagree) 2 3 4 5 (Totally Agree)

**They are able to use tools for a specific purpose.**

*(Example: Do you believe that the animals under your care are able to use a tool in order to get food?)*

1 (Totally disagree)  2  3  4  5 (Totally Agree)

## Learning

*Please indicate your level of agreement with the following statements about the animals of this species:*

**They are able to learn things or learn to do things**

1 (Totally disagree)  2  3  4  5 (Totally Agree)

**They can learn to associate two events distant in time**

*(Example: They associate the sound of a bell with the delivery of food some time later)*

1 (Totally disagree)  2  3  4  5 (Totally Agree)

**They are able to reproduce the actions of another animal by imitation.**

1 (Totally disagree)  2  3  4  5 (Totally Agree)

**After observing others achieve a goal and receive a reward animals under your care can be influenced and attempt to achieve the same reward.**

(*Example: An animal observes one of its conspecifics successfully obtaining food using a particular technique. The animal is then influenced and attempts to reproduce the same technique to achieve the same goal)*

1 (Totally disagree)  2  3  4  5 (Totally Agree)

**They can mimic actions they are already familiar with, but in a context different from their usual.**

(*Example: The animals under your care have developed specific food-seeking behaviours. If placed in a different environment, they will replicate these same behaviours.)*

1 (Totally disagree)  2  3  4  5 (Totally Agree)

**Abstraction**

*Please indicate your level of agreement with the following statements about the animals of this species:*

**They are able to plan their future, to act for their future needs.**

*(Example: They are able to anticipate and setting aside food for their future needs)*

1 (Totally disagree) 2 3 4 5 (Totally Agree)

**They are able to focus on something specific, disregarding the rest, and apply this to other elements that share this same characteristic.**

*(Example: The animals under your care encounter shiny and matte objects in their environment. When they retrieve shiny objects, they receive a reward. Subsequently, they develop an abstraction by focusing solely on shiny objects)*

1 (Totally disagree) 2 3 4 5 (Totally Agree)

**They are able to categorise elements into categories according to their nature, functions and roles.**

*(Example: Animals under your care recognise and categorise a wooden stick and a stone in the category: "tools used to get food")*

1 (Totally disagree) 2 3 4 5 (Totally Agree)

**They are able to see the world from another animal's point of view.**

1 (Totally disagree) 2 3 4 5 (Totally Agree)

**Human-animal relation**

**How frequently do you interact with the animals of the species you care for?** *

I never interact with the animals.

I interact with the animals once a year or less.

I interact with the animals monthly (one to twelve times a year).

I interact with the animals weekly (one or two times per week).

I interact with the animals on a daily basis.

No answer

**What kind of interaction do you have with the animals under your care?** *

I never interact with the animals.

I observe the animals without interacting with them.

I have visual and communicative interaction.

I have physical contact with the animals, but not play and social interactions.

I have physical contact with the animals, including play and social interactions.

For at least part of the animals life, I have been a surrogate parent (hand-rearing, protection, social development)

No answer

**Are all species the same?**

Comparatively rate the following species according to your thoughts on their overall abilities in terms of consciousness. Consider their abilities to feel basic emotions (e.g. pain, cold, hunger, pleasure), complex emotions (e.g. guilt, sadness), to learn, to solve problems, to use tools, to categorize information, and to predict or interpret the emotions of other animals of the same or other species. Please rate the species from lower (1: Has no consciousness) to higher (7: Has consciousness comparable to humans) overal abilities. You may rate several species with the same number. *

| | 1 | 2 | 3 | 4 | 5 | 6 | 7 |
|---|---|---|---|---|---|---|---|
| Chimpanzee (*Pan troglodytes*) | | | | | | | |
| African Elephant (*Loxodonta africana*) | | | | | | | |
| ottlenose Dolphin (*Tursiops truncatus*) | | | | | | | |
| Rat (*Rattus norvegicus*) | | | | | | | |
| Leopard (*Panthera pardus*) | | | | | | | |
| Wolf (*Canis lupus*) | | | | | | | |
| Ball python (*Python regius*) | | | | | | | |
| Raven (*Corvus corax*) | | | | | | | |
| Atlantic Salmon (*Salmo salar*) | | | | | | | |
| Common Octopus (*Octopus vulgaris*) | | | | | | | |
| Honey Bee (*Apis mellifera*) | | | | | | | |
| House fly (*Musca domestica*) | | | | | | | |
| Tree Frog (*Agalychnis callidryas*) | | | | | | | |
| Cockroach (*Blattella germanica*) | | | | | | | |
| House sparrow (*Passer domesticus*) | | | | | | | |
