# Supplementary material for: A Global Survey on the Perception of Conservationists Regarding Animal Consciousness
Source: Animals (Basel). 2025 Jan 24;15(3):341. doi: 10.3390/ani15030341 (PMC11816229; doi:10.3390/ani15030341)
Supplement: Supplementary file 1 [file animals-15-00341-s001.zip › Table S1.pdf]

Table S1. Summary of dimensions, investigation methods, corresponding questions, and examples used in the survey ([1]Questions and examples originally within the dimension "Self-consciousness").

Dimension	Investigation methods	Survey's affirmation about animals	Example
Perceptual Richness	▪ Perceptual categorization<br>▪ Cross-modal learning<br>▪ Discrimination learning	They can recognize elements in their surroundings and categorize them	They see a keeper and are able to classify him into the category "Human", or they see an insect, a fruit, a bottle of milk or a meat, and classify it into the category "Food"
		The animals of this species are able to react differently to a stimulus, depending on whether it's a rewarded stimulus or an unrewarded one	Animals might react differently to a husbandry cue depending on whether you are holding a food reward item
Evaluative Richness	▪ Motivational trade-off (behaviour)<br>▪ Play<br>▪ Judgement bias<br>▪ Preference testing	They can perceive and evaluate their own internal or external environments, and respond accordingly	They perceive the change in temperature when they arrive in a cold or hot place, and they perceive the change in their emotions when they move from well-being to sadness
		They can endure uncomfortable stimuli to get something they like	They would be able to choose to endure electric shock or extreme temperature (very cold or very hot) to eat their favourite food
		They can have preferences and attribute different values to different items or experiences	The animals under your care are capable of attributing different values to different food items, rewards or enrichments, and will work harder to obtain those they prefer

Integration at a time	▪ Interocular transfer<br>▪ Multi-sensory integration<br>Meta-control	They perceive the experiences they live in a unifying way	An animal in search of food perceives the steps to get there—its movements, its discretion, its search for food or its hunt—as a continuous action and not as distinct events
		They can assimilate information that comes from various senses at the same time	They can see the movement of a bouncing ball and understand that the sound they hear at the same time is that of the ball. The same would apply for scents, tactile experiences in addition to acoustic and visual stimuli
Integration across time	▪ Apparent motion<br>▪ Flicker-fusion threshold<br>▪ Memory of objects and events	They can remember events that happened to them in the past [1]	They can remember the loss of a loved one, or where they stored food [1]
		They perceive the world and the events in an uninterrupted way	Do you think that the animals under your care perceive the world continuously, without interruption, while reacting to changes in their environment, and not as unrelated momentary events?
Self-consciousness	▪ Bodily/Body awareness<br>▪ Mirror self-recognition<br>▪ Mental time travel	They can understand that their own body prevents them from successfully resolving a problem	They can understand that their size prevents them from passing through a narrow path
		They can recognize themselves in a mirror (as distinct from another individual of the same or other species)	

Experience of Agency	▪ Response-inhibition<br>▪ Delayed gratification tests	They can understand when they cannot directly achieve a reward and therefore implement indirect strategies to achieve it	Animals are placed in front of a transparent box with food inside that they cannot reach directly. On the side of this box there is a small trap that they can open to reach the food. Animals can understand it and reach the food through this trap
		They can forgo an immediate reward to obtain a better one in the long term	You present a piece of food to an animal but show a favourite food item in your hand. It can either take the food or wait to receive the preferred food later. The animal is capable of understanding and waiting for the preferred food
Experience of Ownership	▪ Body-world discrimination<br>▪ Rubber-hand or rubber-tail illusions	They perceive their body parts as their own	
		They consider a rubber-hand or rubber-tail as their own hand or tail	The animals will recognize or react to a replica of one of their body parts (placed in proximity to the real part) as if it were their own
Reasoning	▪ Transitive inference<br>▪ Mindreading<br>▪ Metacognition<br>▪ Tool-use	They can infer logical conclusions from initial information	When an animal notices the surroundings getting darker (initial information), it will seek shelter to protect itself from a possible danger
		They are capable of consciously deducing the thoughts, mental states, desires, beliefs and even the goals of others	Two animals face each other separated by a barrier. Only one of them receives food. The one who has received food is able to deduce that the other desires to eat and hopes to receive food also
		They can recognize that the actions of others are influenced by their beliefs	An animal is presented with an object that is then hidden under a cup in its presence. When the animal is absent, the object is moved under another cup. Another animal observes the scene. He knows the new location of the object and instinctively looks at the initial cup when the first animal returns. He anticipates its counterpart's false belief, knowing that the first animal thinks the object is still at its initial position
		They can use tools for a specific purpose	Do you believe that the animals under your care are able to use a tool to get food?

Learning	▪ Trace conditioning<br>▪ Reversal learning<br>▪ Imitation learning<br>▪ One trial learning	They can learn things or learn to do things	
		They can learn to associate two events distant in time	They associate the sound of a bell with the delivery of food sometime later
		They can reproduce the actions of another animal by imitation	
		After observing others achieve a goal and receive a reward animal under your care can be influenced and attempt to achieve the same reward.	An animal observes one of its conspecifics successfully obtaining food using a particular technique. The animal is then influenced and attempts to reproduce the same technique to achieve the same goal
		They can mimic actions they are already familiar with, but in a context different from their usual	The animals under your care have developed specific food-seeking behaviours. If placed in a different environment, they will replicate these same behaviours
Abstraction	▪ Conceptual categorization<br>▪ Perspective-taking	They can plan their future, to act for their future needs [1]	They can anticipate and set aside food for their future needs[1]
		They can focus on something specific, disregarding the rest, and apply this to other elements that share this same characteristic	The animals under your care encounter shiny and matte objects in their environment. When they retrieve shiny objects, they receive a reward. Subsequently, they develop an abstraction by focusing solely on shiny objects
		They can categorise elements into categories according to their nature, functions and roles	Animals under your care recognise and categorise a wooden stick and a stone in the category: “tools used to get food”
		They can see the world from another animal’s point of view	
