# Supplementary material for: A Global Survey on the Perception of Conservationists Regarding Animal Consciousness
Source: Animals (Basel). 2025 Jan 24;15(3):341. doi: 10.3390/ani15030341 (PMC11816229; doi:10.3390/ani15030341)
Supplement: Supplementary file 1 [file animals-15-00341-s001.zip › Table S2.pdf]

**Table S2.** Demographic characterization of responses

| Variable | Level | n | % |
|---|---|---|---|
| Country of residence * | Africa | 8 | 8.70 |
| | America | 26 | 28.26 |
| | Asia | 3 | 3.26 |
| | Europe | 52 | 56.52 |
| | Oceania | 3 | 3.26 |
| | Total (N) | 92 | 100 |
| Nationality * | Africa | 7 | 7.61 |
| | America | 22 | 23.91 |
| | Asia | 6 | 6.52 |
| | Europe | 50 | 54.35 |
| | Oceania | 4 | 4.35 |
| | Multiple | 3 | 3.26 |
| | Total (N) | 92 | 100 |
| Age | 18-24 | 6 | 6.52 |
| | 25-34 | 35 | 38.04 |
| | 35-44 | 26 | 28.26 |
| | 45-54 | 16 | 17.39 |
| | 55-64 | 6 | 6.52 |
| | 65-74 | 3 | 3.26 |
| | Total (N) | 92 | 100 |
| Gender | Feminine | 56 | 60.87 |
| | Masculine | 36 | 39.13 |
| | Total (N) | 92 | 100 |
| Level of education | BSc | 27 | 29.35 |
| | MSc | 31 | 33.70 |
| | PhD | 19 | 20.65 |
| | Primary and secondary education | 15 | 16.30 |
| | Total (N) | 92 | 100 |
| Profession | Biologist, ecologist and conservationist | 32 | 34.78 |
| | Ethologist | 2 | 2.17 |
| | Keeper, caretaker and rehabilitator | 24 | 26.09 |
| | Other | 3 | 3.26 |
| | Veterinarian | 27 | 29.35 |
| | Veterinary technician and nurse | 4 | 4.35 |
| Work time duration | Minimum | Maximum | Average |
| | 0 | 52 | 9.96 ±9.23 |
