# Supplementary material for: A Global Survey on the Perception of Conservationists Regarding Animal Consciousness
Source: Animals (Basel). 2025 Jan 24;15(3):341. doi: 10.3390/ani15030341 (PMC11816229; doi:10.3390/ani15030341)
Supplement: Supplementary file 1 [file animals-15-00341-s001.zip › Table S3.pdf]

Table S3. Distribution of the country of residence of the participants

Country of residence	n
Argentina	2
Australia	2
Austria	4
Belize	1
Botswana	1
Brazil	6
Bulgaria	1
Canada	2
Cayman Islands	1
Chile	1
Costa Rica	1
France	24
Germany	1
India	3
Ireland	1
Kenya	1
Kosovo	2
Namibia	1
New Zealand	1
Panama	1
Portugal	10
Prishtin	1
South Africa	5
Spain	6
UK	2
USA	10
Uruguay	1
Total (N)	92
