# Supplementary material for: A Global Survey on the Perception of Conservationists Regarding Animal Consciousness
Source: Animals (Basel). 2025 Jan 24;15(3):341. doi: 10.3390/ani15030341 (PMC11816229; doi:10.3390/ani15030341)
Supplement: Supplementary file 1 [file animals-15-00341-s001.zip › Table S4.pdf]

Table S4 Distribution of the nationality of the participants

Nationality	n
Albanian	2
American	7
Argentinian	2
Australian	3
Austrian	3
Belgian	1
Brazilian	6
British	1
Bulgarian	1
Canadian	2
Caucasian	2
Chilean	1
Costa Rican	1
French	22
German	2
Indian	3
Irish	1
Israeli	1
Italian	1
Jamaican	1
Kenyan	1
Motswana	1
New Zealander	1
Panamenian	1
Portuguese	10
Several	3
South African	5
Spanish	4
Swiss	1
Uruguayan	1
Kosovar	1
Total (N)	92
