# Supplementary material for: A Global Survey on the Perception of Conservationists Regarding Animal Consciousness
Source: Animals (Basel). 2025 Jan 24;15(3):341. doi: 10.3390/ani15030341 (PMC11816229; doi:10.3390/ani15030341)
Supplement: Supplementary file 1 [file animals-15-00341-s001.zip › Table S5.pdf]

Table S5. Taxonomic class, order and family of the animals participants chose to respond about (responses marked with "*" were excluded from further statistical analysis, as they did not focus on a single species)

Class	Ordem	Family
Actinopterygii (n=1, 1.09%)	Anguilliformes (n=1, 1.09%)	Anguillidae (n=1, 1.09%)
Aves (n=24, 26.09%)	Accipitriformes (n=8, 8.70%)	Accipitridae (n=7, 7.61%)
		Pandionidae (n=1, 1.09%)
	Apodiformes (n=1, 1.09%)	Apodidae (n=1, 1.09%)
	Charadriiformes (n=2, 2.17%)	Laridae (n=2, 2.17%)
	Falconiformes (n=1, 1.09%)	Falconidae (n=1, 1.09%)
	Galliformes (n=1, 1.09%)	Phasianidae (n=1, 1.09%)
	Passeriformes (n=2, 2.17%)	Ploceidae (n=1, 1.09%)
		Dasyornithidae (n=1, 1.09%)
	Phoenicopteriformes (n=1, 1.09%)	Phoenicopteridae (n=1, 1.09%)
	Psittaciformes (n=1, 1.09%)	Strigopidae (n=1, 1.09%)
	Sphenisciformes (n=2, 2.17%)	Spheniscidae (n=2, 2.17%)
	Strigiformes (n=4, 4.35%)	Tytonidae (n=2, 2.17%)
		Strigidae (n=2, 2.17%)
	Suliformes (n=1, 1.09%)	Sulidae (n=1, 1.09%)
Cephalopoda (n=1, 1.09%)	Octopoda (n=1, 1.09%)	Octopodidae (n=1, 1.09%)
Chondrichthyes (n=1, 1.09%)	Carcharhiniformes (n=1, 1.09%)	Sphyrnidae (n=1, 1.09%)
Insecta (n=1, 1.09%)	Odonata (n=1, 1.09%)	Lestidae (n=1, 1.09%)
Mammalia (n=47, 51.09%)	Artiodactyla (n=6, 6.52%)	Balaenopteridae (n=1, 1.09%)
		Bovidae (n=2, 2.17%)
		Cervidae (n=1, 1.09%)
		Delphinidae (n=1, 1.09%)
		Giraffidae (n=1, 1.09%)
	Carnivora (n=19, 20.65%)	Felidae (n=8, 8.70%)
		Canidae (n=2, 2.17%)
		Ursidae (n=9, 9.78%)
	Chiroptera (n=1, 1.09%)	Vespertilionidae (n=1, 1.09%)
	Eulipotyphla (n=5, 5.43%)	Erinaceidae (n=5, 5.43%)
	Perissodactyla (n=4, 4.35%)	Rhinocerotidae (n=1, 1.09%)
		Equidae (n=3, 3.26%)
	Pilosa (n=1, 1.09%)	Choloepodidae (n=1, 1.09%)
	Primates (n=6, 6.52%)	Atelidae (n=3, 3.26%)
		Hominidae (n=3, 3.26%)
	Proboscidea (n=4, 4.35%)	Elephantidae (n=4, 4.35%)
	Rodentia (n=1, 1.09%)	Caviidae (n=1, 1.09%)
Reptilia (n=12, 13.04%)	Crocodilia (n=2, 2.17%)	Gavialidae (n=1, 1.09%)
		Crocodylidae (n=1, 1.09%)
	Squamata (n=3, 3.26%)	Elapidae (n=2, 2.17%)
		Iguanidae (n=1, 1.09%)
	Testudines (n=7, 7.61%)	Cheloniidae (n=4, 4.35%)
		Podocnemididae (n=1, 1.09%)
		Emydidae (n=1, 1.09%)
		Dermochelyidae (n=1, 1.09%)
	Several * (n=5, 5.43%)	
