# Supplementary material for: A Global Survey on the Perception of Conservationists Regarding Animal Consciousness
Source: Animals (Basel). 2025 Jan 24;15(3):341. doi: 10.3390/ani15030341 (PMC11816229; doi:10.3390/ani15030341)
Supplement: Supplementary file 1 [file animals-15-00341-s001.zip › Table S6.pdf]

Table S6. Distribution of the averages and STDEV obtained for each question on animal consciousness

Questions	Average	STDEV	n
Q28	1.89	1.08	82
Q15	2.16	1.02	79
Q18	2.27	1.28	81
Q19	2.30	1.41	86
Q11	2.52	1.27	82
Q25	2.65	1.46	84
Q13	2.68	1.34	84
Q27	2.77	1.45	83
Q26	2.94	1.36	82
Q4	3.07	1.30	87
Q17	3.08	1.40	84
Q12	3.29	1.37	84
Q23	3.34	1.39	86
Q22	3.36	1.46	86
Q10	3.62	1.27	85
Q24	3.65	1.26	86
Q9	3.76	1.08	83
Q5	4.05	1.22	87
Q8	4.07	1.15	86
Q21	4.08	1.25	85
Q2	4.09	1.20	86
Q20	4.14	1.19	86
Q14	4.15	1.05	86
Q6	4.17	0.97	86
Q7	4.34	0.89	87
Q16	4.36	0.88	87
Q3	4.43	0.88	87
Q1	4.50	0.85	86
