# Supplementary material for: A Global Survey on the Perception of Conservationists Regarding Animal Consciousness
Source: Animals (Basel). 2025 Jan 24;15(3):341. doi: 10.3390/ani15030341 (PMC11816229; doi:10.3390/ani15030341)
Supplement: Supplementary file 1 [file animals-15-00341-s001.zip › Table S7.pdf]

Table S7 Distribution of the averages and STDEV obtained for each dimension of animal consciousness.

**Dimensions of animal consciousness from which questions are derived**	**Average**	**STDEV**
Abstraction (Q25, Q26, Q27, Q28)	2.56	1.40
Experience of Agency (Q12, Q13)	2.98	1.38
Reasoning (Q16, Q17, Q18, Q19)	3.00	1.52
Self-consciousness (Q10, Q11)	3.07	1.38
Experience of Ownership (Q14, Q15)	3.16	1.43
Learning (Q20, Q21, Q22, Q23, Q24)	3.71	1.35
Evaluative-Richness (Q3, Q4, Q5)	3.85	1.28
Integration across time (Q8, Q9)	3.91	1.12
Integration at a time (Q6, Q7)	4.26	0.93
Perceptual-Richness (Q1, Q2)	4.30	1.06
