# Supplementary material for: A Global Survey on the Perception of Conservationists Regarding Animal Consciousness
Source: Animals (Basel). 2025 Jan 24;15(3):341. doi: 10.3390/ani15030341 (PMC11816229; doi:10.3390/ani15030341)
Supplement: Supplementary file 1 [file animals-15-00341-s001.zip › Table S8.pdf]

Table S8. Distribution of the averages and STDEV obtained for each dimension of animal consciousness from which questions are derived, according to taxonomic classification.

AVES (n=24)		
**Dimensions of animal consciousness**	**Average**	**STDEV**
Experience of Agency	2.50	1.03
Abstraction	2.53	1.26
Self-consciousness	2.83	1.17
Reasoning	2.90	1.34
Experience of Ownership	3.10	1.21
Learning	3.45	1.26
Evaluative-Richness	3.56	1.30
Integration across time	3.71	1.01
Perceptual-Richness	4.02	1.12
Integration at a time	4.13	0.91

AVES (n=24)			
**Order**	**Dimensions of animal consciousness**	**Average**	**STDEV**
Accipitriformes (n=8)	Abstraction	2.28	1.22
	Experience of Agency	2.38	1.09
	Reasoning	2.88	1.41
	Self-consciousness	3.13	1.20
	Experience of Ownership	3.13	1.09
	Evaluative-Richness	3.54	1.38
	Learning	3.65	1.27
	Integration across time	3.94	0.85
	Integration at a time	4.19	0.98
	Perceptual-Richness	4.37	1.17
Galliformes (n=1)	Abstraction	1.75	0.96
	Experience of Agency	2.50	0.71
	Self-consciousness	3.00	1.41
	Experience of Ownership	3.00	1.41
	Reasoning	3.00	1.63
	Integration across time	3.50	0.71
	Learning	3.60	0.55
	Evaluative-Richness	3.67	1.15
	Perceptual-Richness	4.00	1.41
	Integration at a time	4.50	0.71
Passeriformes (n=2)	Abstraction	1.63	0.92
	Learning	2.20	0.63
	Experience of Agency	2.25	0.50
	Reasoning	2.63	1.51
	Self-consciousness	2.75	0.96
	Experience of Ownership	2.75	1.26

	Perceptual-Richness	3.00	1.15
	Integration across time	3.00	0.82
	Evaluative-Richness	3.33	1.37
	Integration at a time	4.25	0.96
Psittaciformes (n=1)	Self-consciousness	3.00	1.41
	Reasoning	3.25	1.26
	Abstraction	3.25	0.96
	Experience of Agency	3.50	0.71
	Experience of Ownership	3.50	2.12
	Integration at a time	4.00	1.41
	Learning	4.20	0.84
	Evaluative-Richness	4.33	1.15
	Integration across time	4.50	0.71
	Perceptual-Richness	5.00	
Sphenisciformes (n=2)	Abstraction	3.00	0.76
	Experience of Agency	3.25	0.50
	Self-consciousness	3.50	0.58
	Reasoning	3.50	1.31
	Evaluative-Richness	3.67	0.82
	Learning	3.70	0.95
	Integration at a time	3.75	0.50
	Experience of Ownership	3.75	0.96
	Integration across time	4.00	
	Perceptual-Richness	4.25	0.96
Strigiformes (n=4)	Self-consciousness	2.63	1.60
	Experience of Agency	2.88	0.83
	Abstraction	2.88	1.41
	Experience of Ownership	3.00	1.31
	Reasoning	3.00	1.10
	Evaluative-Richness	3.50	1.17
	Integration across time	3.50	1.31
	Learning	3.56	0.92
	Perceptual-Richness	3.88	1.13
	Integration at a time	4.13	0.83

**MAMMALIA (n=47)**		
**Dimension of Animal Consciousness**	**Average**	**STDEV**
Abstraction	2.83	1.44
Reasoning	3.21	1.53
Experience of Ownership	3.33	1.52
Self-consciousness	3.39	1.38
Experience of Agency	3.46	1.39
Learning	4.13	1.17
Evaluative-Richness	4.15	1.16
Integration across time	4.31	0.89
Integration at a time	4.39	0.91

Perceptual-Richness	4.56	0.95

**MAMMALIA (n=47)**			
**Order**	**Dimensions of animal consciousness**	**Average**	**STDEV**
Artiodactyla (n=6)	Abstraction	2.86	1.36
	Experience of Agency	3.17	1.53
	Reasoning	3.43	1.41
	Self-consciousness	3.50	1.43
	Experience of Ownership	3.50	1.27
	Evaluative-Richness	4.06	1.11
	Learning	4.47	0.86
	Integration at a time	4.50	0.67
	Integration across time	4.50	0.52
	Perceptual-Richness	4.67	0.78
Carnivora (n=19)	Abstraction	2.94	1.47
	Reasoning	3.01	1.61
	Experience of Agency	3.37	1.30
	Self-consciousness	3.42	1.24
	Experience of Ownership	3.47	1.52
	Learning	4.13	1.21
	Evaluative-Richness	4.21	1.18
	Integration at a time	4.29	1.04
	Integration across time	4.39	0.89
	Perceptual-Richness	4.63	0.97
Perissodactyla (n=4)	Experience of Ownership	2.67	1.97
	Abstraction	3.10	1.45
	Self-consciousness	3.33	1.37
	Experience of Agency	3.50	1.73
	Reasoning	3.79	1.37
	Evaluative-Richness	4.25	1.36
	Perceptual-Richness	4.38	0.92
	Integration across time	4.43	0.79
	Learning	4.45	1.00
	Integration at a time	4.75	0.46
Proboscidea (n=4)	Abstraction	3.19	1.38
	Experience of Ownership	3.50	1.51
	Reasoning	3.56	1.36
	Integration across time	4.13	1.13
	Integration at a time	4.25	1.04
	Learning	4.30	1.26
	Evaluative-Richness	4.33	0.65
	Self-consciousness	4.38	0.92
	Experience of Agency	4.63	0.74
	Perceptual-Richness	5.00	

Primates (n=6)	Experience of Ownership	3.33	1.44
	Abstraction	3.42	1.32
	Evaluative-Richness	4.00	1.28
	Self-consciousness	4.00	1.21
	Reasoning	4.00	1.10
	Experience of Agency	4.08	1.24
	Integration across time	4.09	1.04
	Learning	4.30	0.88
	Integration at a time	4.58	0.79
	Perceptual-Richness	4.75	0.45
**Familly**			
Canidae (n=2)	Experience of Ownership	3.50	1.29
	Integration at a time	3.75	0.50
	Self-consciousness	4.00	1.15
	Experience of Agency	4.00	1.15
	Abstraction	4.00	1.51
	Reasoning	4.13	0.99
	Integration across time	4.25	1.50
	Evaluative-Richness	4.33	1.03
	Perceptual-Richness	5.00	
	Learning	5.00	
Felidae (n=8)	Abstraction	2.55	1.45
	Reasoning	2.67	1.67
	Experience of Agency	2.94	1.65
	Self-consciousness	3.13	1.41
	Experience of Ownership	3.14	1.70
	Learning	3.85	1.42
	Evaluative-Richness	3.96	1.49
	Integration at a time	4.25	1.39
	Perceptual-Richness	4.33	1.37
	Integration across time	4.56	0.89
Ursidae (n=9)	Abstraction	3.03	1.38
	Reasoning	3.06	1.58
	Self-consciousness	3.56	1.10
	Experience of Agency	3.61	0.85
	Experience of Ownership	3.72	1.45
	Learning	4.18	1.05
	Integration across time	4.28	0.75
	Evaluative-Richness	4.41	0.84
	Integration at a time	4.44	0.70
	Perceptual-Richness	4.78	0.43

REPTILIA (n=12)		
**Dimension of Animal Consciousness**	**Average**	**STDEV**
Abstraction	1.57	1.15
Experience of Agency	2.09	1.44
Self-consciousness	2.36	1.43
Reasoning	2.53	1.69
Learning	2.57	1.59
Integration across time	2.86	1.42
Experience of Ownership	2.91	1.51
Evaluative-Richness	3.47	1.50
Perceptual-Richness	3.82	1.22
Integration at a time	3.92	1.06

REPTILIA (n=12)			
**Order**	**Dimensions of animal consciousness**	**Average**	**STDEV**
Crocodilia (n=2)	Abstraction	2.38	1.77
	Self-consciousness	3.50	1.91
	Experience of Ownership	3.50	1.91
	Integration across time	3.75	1.50
	Experience of Agency	3.75	1.89
	Learning	3.90	1.52
	Evaluative-Richness	4.17	1.60
	Reasoning	4.25	1.49
	Perceptual-Richness	4.75	0.50
	Integration at a time	4.75	0.50
Testudines (n=7)	Abstraction	1.50	1.00
	Experience of Agency	1.86	1.17
	Reasoning	2.18	1.47
	Self-consciousness	2.29	1.27
	Learning	2.57	1.56
	Integration across time	2.93	1.38
	Experience of Ownership	3.15	1.46
	Evaluative-Richness	3.62	1.47
	Perceptual-Richness	3.75	1.36
	Integration at a time	3.93	1.07
Squamata (n=3)	Abstraction	1.00	
	Experience of Agency	1.25	0.50
	Learning	1.36	0.50
	Self-consciousness	1.50	1.00
	Integration across time	1.75	0.96
	Experience of Ownership	1.80	0.84
	Reasoning	2.11	1.69

	Evaluative-Richness	2.67	1.32
	Perceptual-Richness	3.33	1.32
	Integration at a time	3.33	1.03
**Familly**			
Elapidae (n=2)	Abstraction	1.00	
	Experience of Agency	1.25	0.50
	Learning	1.40	0.52
	Self-consciousness	1.50	1.00
	Experience of Ownership	1.50	0.58
	Integration across time	1.75	0.96
	Reasoning	1.88	1.64
	Evaluative-Richness	2.83	1.33
	Perceptual-Richness	3.50	1.29
	Integration at a time	3.50	1.29
Iguanidae (n=1)	Learning	1.00	
	Evaluative-Richness	2.33	1.53
	Perceptual-Richness	3.00	
	Integration at a time	3.00	
	Experience of Ownership	3.00	
	Reasoning	4.00	
	Integration across time	NA	
	Self-consciousness	NA	
	Experience of Agency	NA	
	Abstraction	NA	
