# Supplementary material for: A Global Survey on the Perception of Conservationists Regarding Animal Consciousness
Source: Animals (Basel). 2025 Jan 24;15(3):341. doi: 10.3390/ani15030341 (PMC11816229; doi:10.3390/ani15030341)
Supplement: Supplementary file 1 [file animals-15-00341-s001.zip › Table S9.pdf]

Table S9. Distribution of perception index according to the Class.

Class	Average of PI	STDEV	n
Reptilia	71.25	26.15	12
Insecta	83.00		1
Actinopterygii	88.00		1
Aves	89.92	18.12	24
Chondrichthyes	95.00		1
Mammalia	101.38	17.41	47
Cefalopoda	104.00		1
