# Supplementary figures and images for: A Global Survey on the Perception of Conservationists Regarding Animal Consciousness
Source: Animals (Basel). 2025 Jan 24;15(3):341. doi: 10.3390/ani15030341 (PMC11816229; doi:10.3390/ani15030341)

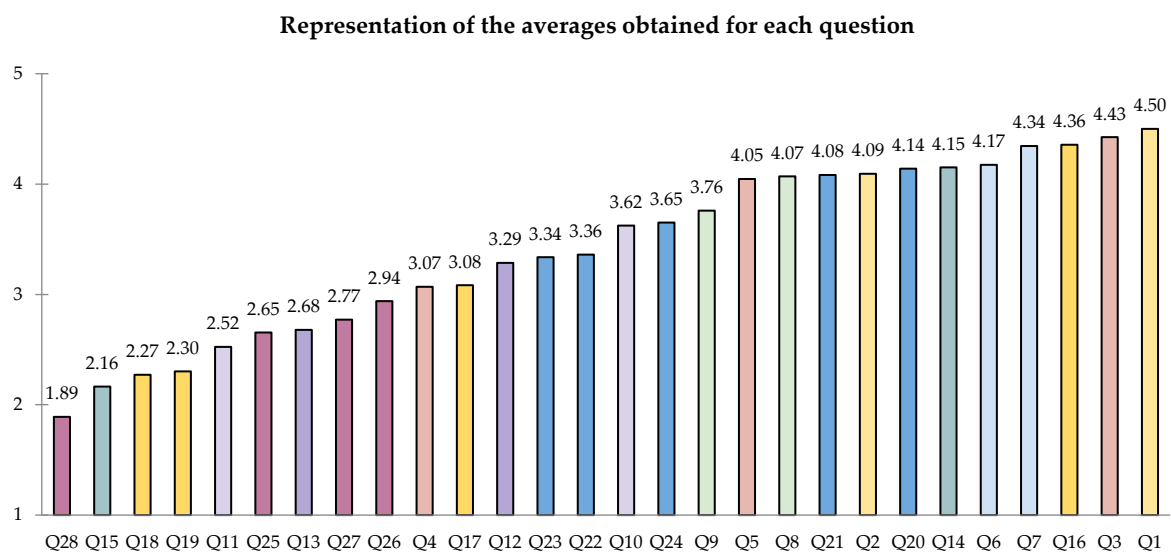

**Figure S2.** Representation of the averages obtained for each question.

Supplement: Supplementary file 1 [file animals-15-00341-s001.zip › Figure S2.pdf]
